# Fecal Butyrate and Deoxycholic Acid Concentrations Correlate With Mortality in Patients With Liver Disease

**DOI:** 10.1016/j.gastha.2025.100695

**Published:** 2025-05-09

**Authors:** Matthew A. Odenwald, Ramanujam Ramaswamy, Huaiying Lin, Christopher Lehmann, Angelica Moran, Michael W. Mullowney, Ashley M. Sidebottom, Antonio Hernandez, Mary McMillin, Amber Rose, David Moran, Jessica Little, Dinanath Sulakhe, Mark D'Souza, Che Woodson, Talha Tanveer, Alexander de Porto, Nicholas Dylla, Anitha Sundararajan, Victoria Burgo, Jackelyn Cantoral, Caroline Jadczak, Emerald Adler, Andrew Aronsohn, Eric G. Pamer, Mary E. Rinella

**Affiliations:** 1 Department of Medicine, Section of Gastroenterology, Hepatology, and Nutrition, University of Chicago, Chicago, Illinois; 2 Duchossois Family Institute, The University of Chicago, Chicago, Illinois; 3 Department of Medicine, Section of Infectious Diseases and Global Health, The University of Chicago, Chicago, Illinois; 4 Department of Pathology, The University of Chicago, Chicago, Illinois; 5 Department of Microbiology, The University of Chicago, Chicago, Illinois

**Keywords:** Microbiome, Metabolomics, Metagenomics, Gut–Liver Axis, Cirrhosis

## Abstract

**Background and Aims:**

The intestinal microbiome produces metabolites, including short chain fatty acids (SCFAs) and secondary bile acids (BAs), that impact host physiology. Loss of intestinal microbiome diversity is associated with cirrhosis progression, but the impact of microbiome-associated metabolites on liver disease remains largely undefined. We aimed to correlate fecal metabolite concentrations with the severity and progression of liver disease.

**Methods:**

In this cross-sectional study, fecal samples from patients hospitalized with liver disease were analyzed by shotgun metagenomic sequencing to determine microbiome compositions and targeted mass spectrometry to quantify SCFAs and BAs. Random survival forest and logistic regression models identified clinical, metagenomic, and metabolomic features associated with rehospitalization and survival.

**Results:**

This cross-sectional study included 24 chronic liver disease, 18 compensated cirrhosis, 225 decompensated cirrhosis and 40 acute-on-chronic liver failure patients and 27 control fecal donors. Microbiome sequencing and metabolite profiling correlated microbial diversity and SCFA and BA concentrations with liver disease severity. Butyrate and deoxycholic acid (DCA) were more important features than individual microbial species in random survival forest models predicting 30-day transplant-free survival, and low butyrate and DCA were associated with 30-day mortality (*P* < .0001). After controlling for model for end stage liver disease (MELD)-sodium score, disease stage, age and gender, low fecal concentrations of butyrate and DCA remained significant risk factors for death (Cox 1.38, *P* = .027). Bacterial species associated with butyrate and DCA concentrations included *Bifidobacterium spp.* and *F. prausnitzii*.

**Conclusion:**

Mass spectrometry rapidly identifies patients with low fecal butyrate and DCA concentrations who are at increased risk of 30-day mortality. These findings set the stage for clinical trials of microbiome reconstitution with butyrate and DCA-producing bacterial species.

## Introduction

The intestinal microbiome impacts immune development and epithelial integrity by producing metabolites that are absorbed from the gut and circulated to host tissues. Microbiome compositions and metabolite production vary between individuals and are impacted by environmental exposures, diet, host genetics, inflammatory diseases, and medical treatments, in particular antibiotic administration. Following absorption, microbiome-derived metabolites flow to the liver and bathe hepatocytes and liver-dwelling mesenchymal and immune cells. Clinical studies have demonstrated that advanced liver disease is accompanied by loss of the microbiome’s beneficial bacterial species and expansion of potentially pathogenic microbes.^1^, ^2^, ^3^, ^4^, ^5^ Shifts in microbiome composition have been correlated with disease complications such as hepatic encephalopathy,^6^^,^^7^ systemic infections,^4^ the development of acute-on-chronic liver failure (ACLF),^3^ and death.^2^^,^^3^ Transplantation of feces from patients with alcohol-related hepatitis (AH) to mice has demonstrated that microbiome compositions contribute to liver disease severity when challenged with alcohol.^8^^,^^9^ In clinical trials, fecal microbiota transplantation from healthy human donors to patients with liver disease can improve symptoms of hepatic encephalopathy^10^, ^11^, ^12^ and has survival benefit in AH.^13^ A recent study demonstrated that a bacterially produced cytolysin is a prognostic marker for severe AH and a driver of disease in mouse models;^9^ however, mechanistic insights into the microbiome’s impact on liver disease remain limited.

Bacterially derived and modified metabolites modulate microbiome compositions and host physiology. A recent observational study characterizing microbiome compositions and fecal metabolite concentrations in hospitalized liver disease patients demonstrated marked compositional and metabolomic differences between individuals.^4^ Lactulose-driven expansion of Bifidobacterium species was associated with reduced intestinal colonization by antibiotic-resistant pathogens and lower incidences of spontaneous bacterial peritonitis and bacteremia.^4^ Mass spectrometric profiling of fecal metabolites has also revealed correlations between metabolite concentrations and clinical outcomes in patients with COVID-19 respiratory failure^14^ and infections after liver transplantation.^15^

Metabolic pathway analyses suggest that specific microbiome functions, including production of short chain fatty acids (SCFAs) such as butyrate, correlate with disease progression.^3^ Bile acids (BAs) are produced by the host and modified by commensal microbes into immunomodulatory forms. Primary BAs are produced in the liver and delivered into the gut where they are deconjugated by intestinal bacteria and subsequently converted to a variety of secondary BAs by other gut-dwelling bacterial species. Fecal BA profiles in patients with cirrhosis have reduced concentrations of secondary BAs^4^^,^^16^ and SCFAs.^4^^,^^7^^,^^17^ These findings have led to the yet-to-be-tested hypothesis that microbiome-derived metabolites contribute to the course of chronic liver disease. While pathway analyses of microbial genomes provide insight into a microbiome’s capacity to produce specific metabolites, mass spectrometric quantitation of fecal metabolites provides direct evidence of a microbiome’s activity.

Despite strong preclinical evidence in animal models,^8^^,^^9^^,^^18^ abundant observational clinical data,^1^, ^2^, ^3^, ^4^, ^5^^,^^8^^,^^9^^,^^19^, ^20^, ^21^ positive outcomes in small clinical trials of microbiome-targeted therapies^10^, ^11^, ^12^, ^13^^,^^22^, ^23^, ^24^ and mechanistic plausibility, microbiome therapies have yet to be developed for clinical use in patients with liver disease. In addition to challenges with live biotherapeutic design,^25^ studies of microbiome-targeting therapies are limited by the inability to rapidly identify and stratify patients with the spectrum of possible microbiome deficiencies.

To understand the relationship between metagenomic composition, metabolite production, and liver disease progression, we performed paired metagenomic and metabolomic analyses on fecal samples from 307 patients hospitalized with various stages of liver disease. We demonstrate that loss of microbiome diversity and loss of fecal metabolites associate with disease progression and mortality. We show that fecal metabolite concentrations correlate with mortality more strongly than taxonomic diversity, and by quantifying butyrate and the secondary BA deoxycholic acid (DCA), we rapidly identify patients who may benefit from microbiome-targeting therapies.

## Methods

### Study Design

This was a prospective cohort study of consecutive hospitalized adult hepatology patients at a single institution from April 2021 to Mary 2023. This was an extension of a previously published cohort,^4^ and inclusion and exclusion criteria were unchanged. Specifically, subjects were ≥18 years old, provided informed consent (either themselves or by proxy if unable to provide consent), and being treated on the hepatology consult service. Subjects who had prior solid organ transplant or a prior colectomy were excluded. Patients were enrolled as soon as possible upon hospital admission, most within 48 hours. All research was conducted in accordance with both the Declarations of Helsinki and Istanbul, and the study protocol was approved by the University of Chicago Institutional Review Board (IRB21-0327). The healthy donor fecal samples and fecal samples from independent cohort of patients admitted to the medical intensive care unit (MICU) were collected in a similar manner under protocols approved by the University of Chicago Institutional Review Board (Healthy Donors: IRB20-1384; MICU: IRB20-1102). Written informed consent was obtained from all participants or their surrogate decision makers. Participants were not compensated. All subjects who produced at least 1 sample were included in the analyses. Specific samples were chosen for analysis as indicated in each figure.

### Specimen Collection and Storage

After enrollment, an order for stool collection was placed in the electronic medical record. Stool samples were collected by the clinical nursing teams on inpatient wards and intensive care units. After collection, samples were immediately sent to the microbiology lab through the pneumatic tubing system and stored at +4 °C until collection, aliquoting and storage at −80 °C by the research team (within 24 hours of sample production). If able to provide additional samples, fecal samples were collected approximately every 2 days during hospitalization. Samples were collected in a similar manner on rehospitalization up until 1 year postenrollment or until death or transplant. Samples for healthy donors were provided by donors and immediately placed at +4 °C until aliquoting and storage at −80 °C. Samples remained stored at −80 °C until they were processed for metagenomics and metabolomics.

### Clinical Data Collection

Patients were followed prospectively to assess for development of clinical outcomes. Upon enrollment, all patients were given a unique patient ID that was linked to their unique medical record numbers. The unique ID was stored in a REDCap database along with preadmission medications and disease characteristics, which were obtained by a combination of patient/family recollection and verification with medical records when available. Survival and hospital admission data were extracted by chart review.

### Metagenomic Analyses

DNA was extracted using the QIAamp PowerFecal Pro DNA kit (Qiagen). Before extraction, samples were subjected to mechanical disruption using a bead beating method. Briefly, samples were suspended in a bead tube (Qiagen) along with lysis buffer and loaded on a bead mill homogenizer (Fisherbrand, Beadmill). Samples were then centrifuged, and supernatant was resuspended in a reagent that effectively removed inhibitors. DNA was then purified routinely using a spin column filter membrane and quantified using Qubit. Libraries were prepared using 100 ng of genomic DNA using the QIAseq FX DNA library kit (Qiagen). Briefly, DNA was fragmented enzymatically, and desired insert size was achieved by adjusting fragmentation conditions. Fragmented DNA was end repaired and ‘A’s’ were added to the 3′ends to stage inserts for ligation. During ligation step, Illumina compatible Unique Dual Index adapters were attached to the inserts and the prepared library was amplified by polymerase chain reaction. Amplified libraries were purified, and QC was performed using a Tapestation (Agilent). Normalized libraries were sequenced on an Illumina NextSeq 500 or NextSeq 1000/2000 to generate 2x150bp reads. Metagenomic information is publicly available on National Center for Biotechnology Information under BioProject ID PRJNA1184735 and PRJNA912122 (liver disease cohort), BioProject ID PRJNA1134172 and PRJNA842425 (MICU cohort) and BioProject ID PRJNA838648 (healthy donor cohort).

### Metabolomic Analyses

SCFAs (ie butyrate, acetate, and propionate) were derivatized with pentafluorobenzyl bromide and analyzed via negative ion collision-induced gas chromatography mass spectrometry ([–]CI-GC-MS, Agilent 8890).^26^ Ten BAs (ie primary: cholic acid; conjugated primary: glycocholic acid, taurocholic acid; secondary: DCA, lithocholic acid [LCA], isodeoxycholic acid; modified secondary: alloisolithocholic acid [alloisoLCA] and 3-oxolithocholic acid [3-oxoLCA]) were quantified (μg/mL) by negative mode liquid chromatography-electrospray ionization-quadrupole time-of-flight-MS ([–]LC-ESI-QTOF-MS, Agilent 6546). Data analysis was performed using MassHunter Quantitative Analysis software (version B.10, Agilent Technologies) and confirmed by comparison to authentic standards.

Quantitative fecal metabolomic information paired to fecal metagenomic information is publicly available on National Center for Biotechnology Information under BioProject ID PRJNA1184735 and PRJNA912122 (liver disease cohort), BioProject ID PRJNA1134172 and PRJNA842425 (MICU cohort), and BioProject ID PRJNA838648 (healthy donor cohort). Raw data files are publicly available on the MassIVE data repository (IDs: MSV000092750, MSV000092751, MSV000095462, MSV000096501) and MetaboLights (ID: MTBLS5288).

To quantify both butyrate and DCA in one assay, we developed a liquid chromatography–mass spectrometry (LC-MS/MS) method in which fecal material is extracted in 80% methanol solvent containing deuterated internal standards using beadruptor tubes. After centrifugation, the supernatant is derivatized using 3-Nitrophenylhydrazine and 1-Ethyl-3-(3-dimethylaminopropyl)carbodiimide for 30 minutes and directly analyzed by LC-MS/MS for quantities of microbiota-derived metabolites. For optimal separation of isomers, the injected sample is separated on a CORTECS T3 column with a flow rate of 0.350 mL/min and run time of 20 minutes using the Exion LC AD (SCIEX). Analytes are detected using a SCIEX QTRAP 6500 mass spectrometer with an electrospray ionization source. Full details are described elsewhere.^27^

### Statistical Analyses

All statistical analyses were conducted using the R programming language (version 4.2.2). Data were not assumed to be normal, but this was not formally tested. Adjusted *P* values of the tests were considered to be statistically significant for all analyses conducted if *P* ≤ .05. Continuous variables were compared between the groups using Wilcoxon rank-sum test (rstatix::wilcox_test) and multiple test correction were adjusted following the Benjamini-Hochberg method (stats:: p.adjust). Categorical variables were compared using Fisher’s Exact test (stats::fisher.test). Logistic regression (stats::glm) was used to estimate the odds of multiple factors affecting outcomes. Kaplan–Meier curves for survival were generated after stratifying for selected microbiome parameters including alpha-diversity and butyrate and DCA concentrations (survival::Surv, survfit, ggsurvplot).

### Supplemental Methods

Additional detail for metagenomic analysis, statistical methods and modeling, and metabolite profile prediction can be found in the supplemental methods.

## Results

### Patient Population

We enrolled 500 consecutive patients hospitalized with liver disease in a clinical study to determine the impact of microbiome compositions and metabolites on disease progression. Of these 500 patients, 307 produced at least 1 fecal sample during their index hospitalization and were included in analysis. Demographics and disease characteristics at the time of index hospital admission are shown in Table. Of the patients in our study, 24 (7.8%) had chronic liver disease defined as having chronic liver disease without advanced fibrosis or portal hypertension, 18 (5.9%) had compensated cirrhosis defined as having known cirrhosis without previous decompensation, 225 (73.3%) had decompensated cirrhosis, and 40 (13.0%) were classified as acute-on-chronic liver failure (ACLF) upon admission using criteria set forth by The North American Consortia for the Study of End Stage Liver Disease. More advanced disease (decompensated cirrhosis and ACLF) commonly resulted from alcohol use (174 of 265, 65.6%). Patients classified as chronic liver disease were generally hospitalized for non–liver-related reasons, and 13 (54.2%) had chronic congestion from heart failure without advanced fibrosis. Markers of clinical disease severity, including higher MELD-Na scores, presence of clinically significant portal hypertension, and end organ failure, were more common with more advanced disease. Fecal samples from 27 healthy donors (Table) were also collected for comparison.

**Table:** Patient Demographics and Baseline Disease Characteristics

Characteristics	Healthy donor	Chronic	Cirrhosis (compensated)	Cirrhosis (decompensated)	ACLF	*P*
n	27	24	18	225	40	(Analysis excludes healthy donors)
Age (median [IQR])	34.50 [25.75, 42.50]	57.40 [44.25, 64.00]	64.35 [55.22, 68.58]	57.80 [48.40, 66.40]	56.20 [42.88, 62.47]	.176
Sex = Male (%)	10 (38.5)	13 (54.2)	14 (77.8)	127 (56.4)	27 (67.5)	.199
Race (%)						.811
African American	2 (7.7)	12 (50.0)	6 (33.3)	71 (31.6)	13 (32.5)	
Asian/Pacific Islander	4 (15.4)	1 (4.2)	1 (5.6)	9 (4.0)	2 (5.0)	
Caucasian	16 (61.5)	8 (33.3)	10 (55.6)	111 (49.3)	21 (52.5)	
Hispanic	4 (15.4)	2 (8.3)	1 (5.6)	31 (13.8)	4 (10.0)	
Other	0 (0.0)	1 (4.2)	0 (0.0)	3 (1.3)	0 (0.0)	
BMI (median [IQR])	24.51 [21.07, 27.43]	26.74 [23.76, 33.02]	30.71 [27.07, 33.42]	27.25 [23.34, 33.11]	30.50 [23.20, 34.75]	.34
Liver disease stage (%)						<.001
Chronic	N/A	24 (100.0)	0 (0.0)	0 (0.0)	0 (0.0)	
Cirrhosis (compensated)	N/A	0 (0.0)	18 (100.0)	0 (0.0)	0 (0.0)	
Cirrhosis (decompensated)	N/A	0 (0.0)	0 (0.0)	225 (100.0)	0 (0.0)	
ACLF (NACSELD criteria)	N/A	0 (0.0)	0 (0.0)	0 (0.0)	40 (100.0)	
Primary disease etiology (%)						<.001
Alcohol	N/A	4 (16.7)	3 (16.7)	142 (63.1)	29 (72.5)	
Metabolic dysfunction (MASLD/MASH)	N/A	1 (4.2)	6 (33.3)	32 (14.2)	5 (12.5)	
Vascular and Congestive	N/A	13 (54.2)	3 (16.7)	7 (3.1)	0 (0.0)	
HBV	N/A	0 (0.0)	0 (0.0)	2 (0.9)	1 (2.5)	
HCV	N/A	0 (0.0)	1 (5.6)	16 (7.1)	3 (7.5)	
Autoimmune	N/A	0 (0.0)	1 (5.6)	9 (4.0)	0 (0.0)	
PBC	N/A	0 (0.0)	0 (0.0)	2 (0.9)	0 (0.0)	
PSC	N/A	2 (8.3)	1 (5.6)	5 (2.2)	0 (0.0)	
DILI	N/A	0 (0.0)	0 (0.0)	2 (0.8)	0 (0.0)	
Metabolic (Hemachromatosis, Wilsons)	N/A	0 (0.0)	0 (0.0)	2 (0.9)	1 (2.5)	
Other	N/A	4 (16.7)	3 (16.7)	6 (2.7)	1 (2.5)	
Portal hypertension (%)	N/A	2 (8.3)	8 (44.4)	199 (88.4)	37 (92.5)	<.001
Hepatic encephalopathy (%)	N/A	1 (4.2)	0 (0.0)	42 (18.7)	26 (65.0)	<.001
Respiratory failure (%)	N/A	2 (8.3)	1 (5.6)	11 (4.9)	28 (70.0)	<.001
Renal failure (%)	N/A	1 (4.2)	0 (0.0)	11 (4.9)	20 (50.0)	<.001
Shock (%)	N/A	3 (12.5)	2 (11.1)	13 (5.8)	27 (67.5)	<.001
HCC (%)	N/A	1 (4.2)	5 (27.8)	24 (10.7)	4 (10.0)	.096
invSimp (median [IQR])	15.19 [11.42, 17.86]	12.47 [8.94, 15.71]	11.48 [7.17, 15.11]	5.77 [2.37, 11.94]	2.28 [1.19, 6.76]	<.001
Butyrate (micromolar, median [IQR])	12,530.00 [9395.00, 21,190.00]	8845.00 [3615.00, 12,230.00]	7340.00 [4875.00, 23,232.50]	3480.00 [540.00, 8960.00]	605.00 [430.00, 1727.50]	<.001
DCA (micromolar, median [IQR])	940.16 [371.78, 1814.76]	549.96 [40.79, 962.50]	140.58 [15.44, 636.81]	1.81 [0.03, 52.79]	0.27 [0.00, 1.59]	<.001

Patients are grouped by liver disease stage at the time of enrollment. Chronic liver disease patients are with chronic disease without advanced fibrosis or portal hypertension. Compensated cirrhosis is cirrhosis without a prior decompensation event. ACLF upon admission using criteria set forth by The NACSELD. Clinically significant portal hypertension was defined by hepatic venous pressure gradient ≥10 mmHg, characteristic imaging (enlarged portal vein, intra-abdominal varices, splenomegaly or ascites) or clinical features (ascites, varices or HE). Organ failures, including hepatic encephalopathy, renal failure, respiratory failure and shock, are defined per NACSELD.

BMI, body mass index; HBV, hepatitis B virus; HCC, hepatocellular carcinoma; HCV, hepatitis C virus; IQR, interquartile range; MASH, metabolic dysfunction-associated steatohepatitis; MASLD, metabolic dysfunction-associated steatotic liver disease; NACSELD, North American Consortia for the Study of End Stage Liver Disease; PBC, primary biliary cholangitis; PSC, primary sclerosing cholangitis.

Fecal sample microbiome composition and diversity correlate with stage of liver disease and mortality.

**Figure 1:**
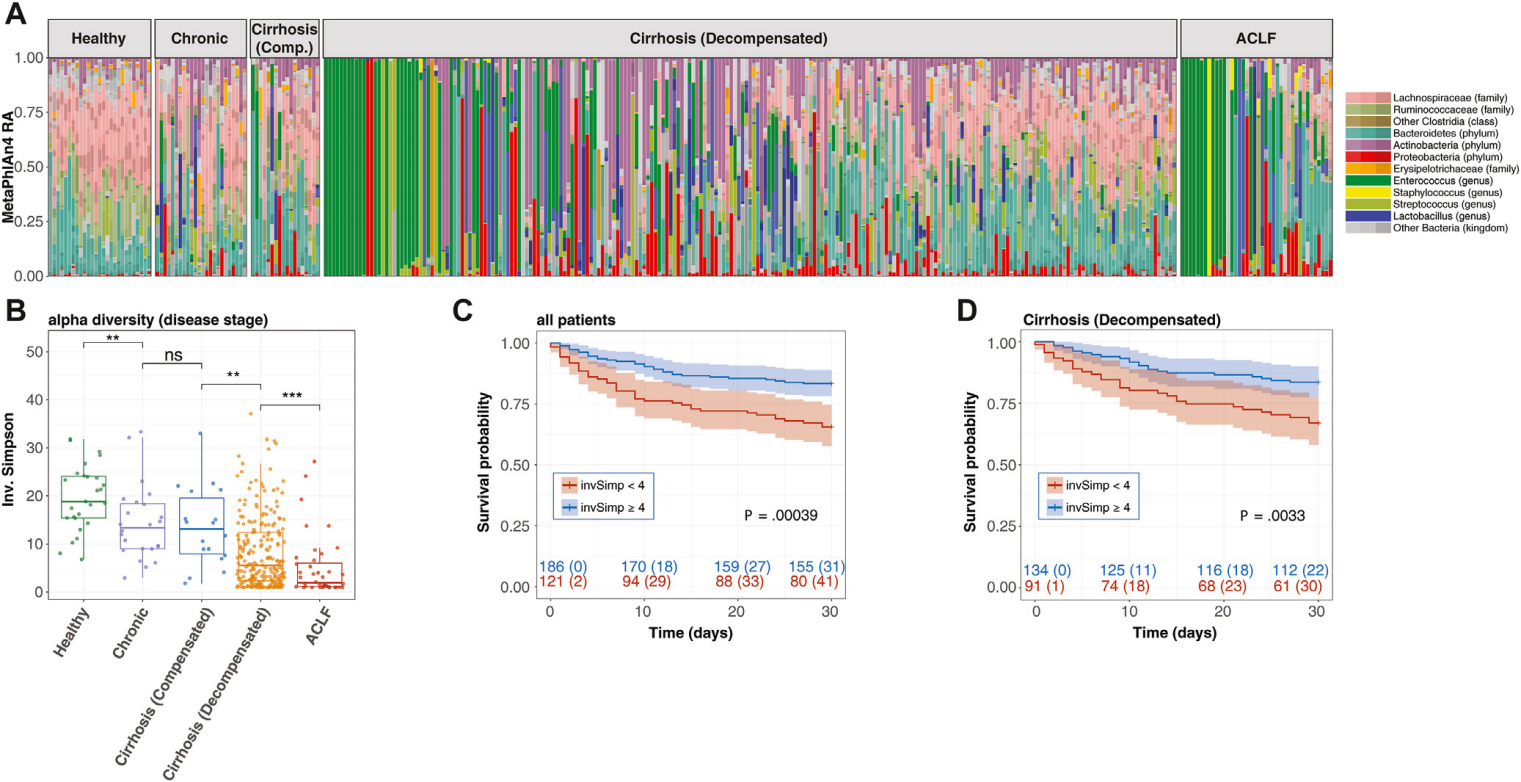
Fecal sample metagenomics correlates with stage of liver disease and mortality. Fecal samples from 307 patients with liver disease and 27 healthy donors were analyzed by shotgun metagenomics. (A) Relative taxa abundance was calculated with MetaPhlan4. Metagenomic alpha-diversity was quantified using the inverse Simpson (invSimp) metric. Samples are arranged in order of increasing invSimp within each group. (B) invSimp values are plotted by liver disease stage. Each point represents a single value. Median and IQR are indicated by the line and box, respectively. Global statistics were done using the Kruskal–Wallis test, and comparisons between individual groups were analyzed using Wilcoxin rank sum. *P*-values shown comparing adjacent disease stages are adjusted for multiple comparisons: ∗, *P* < .05; ∗∗, *P* < .01; ∗∗∗, *P* < .001; ∗∗∗∗, *P* < .0001. (C and D) Kaplan–Meier curves stratified by initial sample alpha-diversity (threshold invSimp = 4) were generated for 30-day transplant-free survival for (C) the entire cohort and (D) only patients with decompensated cirrhosis. The number at risk is shown below. IQR, interquartile range.

Microbiome compositions were determined by shotgun metagenomic sequencing on the initial fecal samples collected from 307 patients with liver disease and 27 healthy donors (Figure 1A). We used MetaPhlAn4 to determine microbiome compositions and inverse Simpson to calculate alpha-diversity. There was a wide range of microbiome compositions and alpha-diversity in patients with liver disease ranging from nearly 100% domination by potentially pathogenic bacterial species such as *Enterococcus faecium* to compositions that were indistinguishable from healthy donors. Alpha-diversity was reduced in patients who progressed to more severe stages of liver disease, particularly in patients with decompensated cirrhosis and patients with ACLF (Figure 1B).

To determine an appropriate threshold, we performed survival analysis on the cohort using incrementally increasing thresholds for invSimp from 1 to 32 (Figure A1). We chose a threshold of invSimp = 4 to differentiate high and low alpha-diversity and demonstrated by 1000 iterations of bootstrapping that observed differences were statistically significant with *P* < .05 (Figure A1). Higher fecal microbiome alpha-diversity (invSimp ≥ 4) on hospital admission was associated with prolonged transplant-free survival in all participants (Figure 1C) and in the subset with decompensated cirrhosis (Figure 1D). These data corroborate previous findings in an independent cohort and suggest that microbiome compositions in patients with liver disease may impact short-term (30 day) mortality.^3^ However, after adjusting for age, sex, MELD-Na and disease stage at enrollment, Cox proportional hazard ratio for invSimp < 4 was 1.24 (*P* = .399) for 30-day transplant free survival. Antibiotic and acid suppression administration within 7 days of sample collection were each associated with reduced fecal alpha-diversity in patients with decompensated cirrhosis (Figure A2A-C), and the associated loss of fecal alpha-diversity paralleled the loss seen with disease progression. When patients were stratified by antibiotic or acid suppression, there was not a significant difference in mortality in the full cohort or in patients with decompensated cirrhosis (Figure A2D-G), and neither medication class was a significant contributor to a Cox-proportional hazard analysis of 30-day survival (antibiotics, Cox HR = −0.18, *P* = .53; acid suppression, Cox HR = −0.018, *P* = .74).

### Fecal Butyrate and DCA Concentrations Correlate with Liver Disease Stage

**Figure 2:**
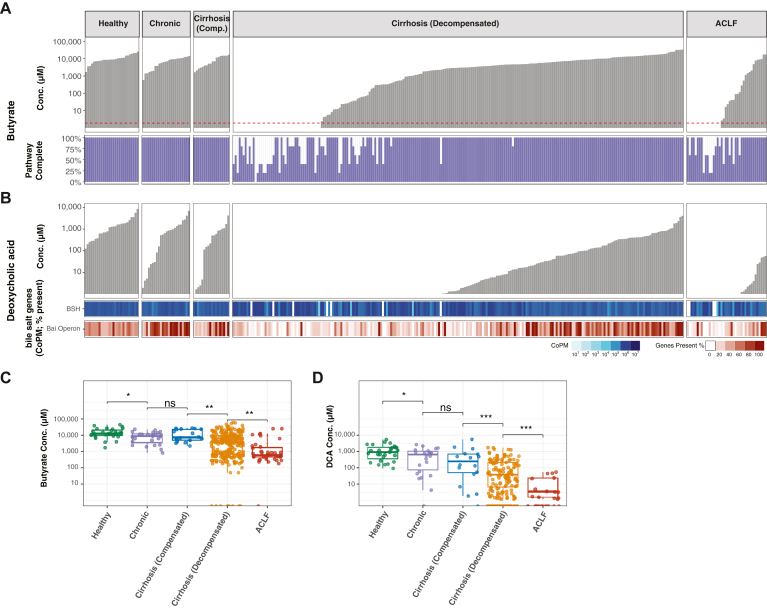
Fecal butyrate and DCA concentrations correlate with liver disease stage. Fecal butyrate and DCA were quantified by LC-MS/MS. Initial fecal samples are grouped by stage of liver disease. Samples are grouped by disease stage and arranged by increasing (A) butyrate or (B) DCA concentrations. Metabolite concentrations (μM) are graphed in the top panels with the red dotted line marking the limit of detection. Bottom panels depict genetic capacity for metabolite generation. (A, bottom) Overall butyrate pathway completeness was calculated as the highest percentage of genes present in any single butyrate production pathway (Figure A3). (B, bottom) Gene copies per megabase were calculated for bile salt hydrolase genes. Percentage of *bai* operon genes detected is shown. (C) Butyrate and (D) DCA concentrations for each liver disease stage are graphed. Median and IQR are indicated by the line and box, respectively. Statistical comparisons between groups were analyzed using the Wilcoxin rank sum. *P*-values shown comparing adjacent disease stages are adjusted for multiple comparisons: ∗, *P* < .05; ∗∗, *P* < .01; ∗∗∗, *P* < .001; ∗∗∗∗, *P* < .0001. IQR, interquartile range.

Microbially produced and modified metabolites impact the host intestinal epithelium and gut-associated immune tissues and systemically; however, our understanding of their production across the spectrum of liver disease is limited. We performed targeted gas chromatography-mass spectrometry and LC-MS to measure concentrations of 14 microbially produced and modified compounds, including 3 SCFAs and 11 primary and secondary BAs. Low alpha-diversity samples had significantly reduced concentrations of SCFAs (butyrate, acetate, and propionate) and secondary and modified secondary BAs and increased concentrations of conjugated primary BAs (Figure 2A and A2). Concentrations of SCFAs correlated with each other, and the secondary BAs DCA and LCA positively correlated with each other and with immunomodulatory modified secondary BAs, such as alloisoLCA, 3-oxoLCA and isodeoxycholic acid (Figure 2B).

Given the correlation between individual SCFAs and between secondary BAs, we selected butyrate and deoxycholate as two representative metabolites. Butyrate impacts enterocyte function and mucosal immunity. DCA is a secondary BA and serves as a precursor to lower abundance bioactive modified secondary BAs. To simultaneously measure butyrate and DCA, we used a combined LC-MS/MS assay.^27^ Similar to alpha-diversity, butyrate and DCA concentrations were lower in patients with more advanced disease (Figure 2). Additionally, antibiotic administration, and to a lesser degree acid suppression exposure, associated with reduced metabolite concentrations in patients with decompensated cirrhosis (Figure A4). Reduced metabolite concentrations correlate with loss of microbial genetically encoded pathways necessary for butyrate production and BA metabolism. Butyrate can be produced by the acetyl-CoA, lysine, glutarate and 4-aminobutyrate pathways, each of which can be completed by multiple complementary genes. At least one pathway was genetically complete in all healthy donors, patients with chronic liver disease and patients with compensated cirrhosis (Figure A5). Patients with genetically complete pathways all had detectable fecal butyrate concentrations. In contrast, 21.2% of patients with decompensated cirrhosis and 35% of patients with ACLF on admission lacked the microbial genetic capacity to produce butyrate and generally had undetectable concentrations of butyrate. Some patients with complete butyrate pathway genes had undetectable or very low butyrate concentrations, possibly reflecting reduced dietary fiber intake or increased butyrate utilization or absorption.

BAs are modified by a series of reactions that include deconjugation via bile salt hydrolases, conversion from primary to secondary BAs by bacterial species that encode the bai operon, and secondary BA modification by bacterial strains encoding hydroxysteroid dehydrogenases (HSDH) and 5-alpha- and 5-beta-reductases. In contrast to bile salt hydrolase genes, which were ubiquitously encoded for in fecal samples from liver disease patients, bai operon genes were coded for more frequently in samples with high levels of secondary BA (Figure 2B). Similarly, bioactive modified secondary BAs, including isodeoxycholic acid, alloisoLCA, and 3-oxoLCA, were found at lower concentrations with more advanced liver disease, and this correlates with HSDH and reductase gene encoding (Figure A6). These results demonstrate that genome analyses largely but incompletely correlate with measured metabolite concentrations, suggesting that metabolite quantitation provides a more functionally relevant perspective on the microbiome’s potential impact on progression of liver disease.

### Microbiome Characteristics at Hospital Discharge and Rehospitalization

To determine whether microbiome compositions or metabolite concentrations correlate with the need for hospital readmission, we analyzed the last fecal sample collected within 3 days before discharge for patients who survived the index hospitalization. Of the 307 patients enrolled in this study who produced a fecal sample, 238 were discharged from the hospital without a liver transplant, and 193 had a fecal sample collected within 3 days of hospital discharge (Figure A7A). Five patients were removed from analysis due to a previous hospitalization. Of these 188 patients, 55 were rehospitalized within 30 days of discharge (Figure A7B). Using Kaplan–Meier analysis, inverse Simpson did not discriminate patients who were rehospitalized within 30 days (Figure A7C). Patients with low butyrate and DCA levels at the time of discharge were more likely to be rehospitalized within 30 days (Figure A7D). However, when adjusting for age, sex, and MELD-Na score, Cox proportional hazards for 30-day readmission were not significant with ratios of 0.915 (*P* = .766) and 0.641 (*P* = .235) for invSimp (reference: invSimp ≥ 4) and metabolite grouping (reference: butyrate and DCA high), respectively.

### Reduced Fecal Butyrate and DCA Concentrations Associate with Increased Short-term Mortality

**Figure 3:**
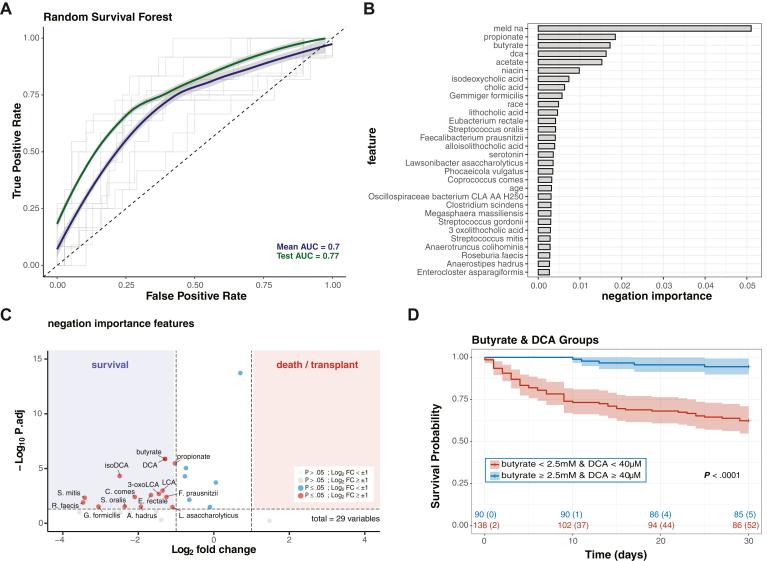
Rapid quantification of fecal butyrate and DCA correlates with 30-day survival better than metagenomic data. A RSF model was generated to assess metagenomic, metabolomic, clinical (laboratory and medication exposures) and demographic features that may contribute to survival. (A) Receiver operator curve for the RSF model of 30-day survival. (B) The top 30 negation important features for RSF model in order of decreasing importance. (C) The most distinguishing features are plotted on a volcano plot (log_2_fold change vs log_10_*P* value) comparing initial samples from surviving (blue) and deceased/transplanted (red) patients. *P* values are corrected for multiple comparisons. Values with log2 fold-change > 1 (corresponding to a 2-fold change) with a *P* < .05 were considered significant. (D) Kaplan–Meier curves stratified by initial sample metabolite concentrations with number at risk shown below. RSF, random survival forest.

To determine whether microbiome characteristics correlate with short-term (30-day) transplant-free survival, we applied a random survival forest model to see which clinical data (including demographics, laboratory measurements, and medication exposures), metagenomic, and metabolomic characteristics best predict 30-day vital status. This produced a model with 77% accuracy in the test set (Figure 3A), with features that contributed to this model shown in Figure 3B. MELD-Na score was the largest contributor predicting 30-day mortality in this patient population, and a model removing MELD-Na at a 67% accuracy in the test set. From microbiome-related features, metabolites, including butyrate and DCA, were more impactful in this model of 30-day mortality than the abundance of any microbial species. The important microbial species included many butyrate producers (e.g. *F. prausnitzii*, *E. rectale*, *C. comes*, and *A. hadrus*) and *C. scindens*, an organism that converts primary to secondary BA. To determine directionality of these contributions, important features were plotted on a volcano plot (Figure 3C), which demonstrated increased levels of bioactive metabolites, including butyrate and DCA, as well as increased levels of commensal organisms associated with SCFA generation and BA metabolism in patients who survived. Grouping patients as either having high or low butyrate/DCA concentrations, Kaplan–Meier analysis showed that patients with high concentrations had substantially improved 30-day survival (>90%) compared to those with low concentrations (68.8%) (Figure 3D and A8). When controlling for MELD-Na, stage of liver disease, age and sex with a Cox proportional hazard model, metabolite levels remained significantly associated with 30-day survival. Cox proportional hazard for 30-day mortality for initial sample metabolite grouping was 1.38 (*P* = .027). The association between low butyrate and DCA by a rapid LC-MS/MS assay and poor outcomes will require an interventional trial to assess causation.

### Butyrate and DCA Quantification Specify the Loss of Beneficial Commensal Microbes

**Figure 4:**
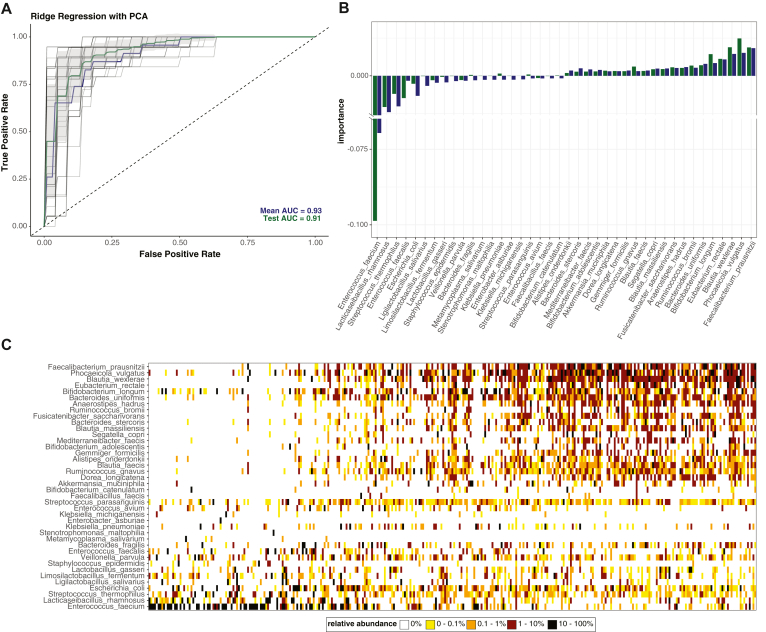
Rapid metabolite measurement aids in rational microbiome therapy design. (A) Receiver operator curve for ridge regression model using species level taxonomy to distinguish between samples above and below metabolite thresholds (butyrate ≥ 2.5 mM and DCA ≥ μM). (B) The importance of 40 species (top and bottom 20 species) to the Ridge regression model is graphed for training (blue) and testing (green). (C) The initial fecal sample from 307 patients with liver disease are arranged in order of increasing butyrate concentrations from left to right, and the relative abundance of the important species from Ridge regression are plotted as a heatmap.

The microbiome can be targeted in clinical trials by administering select commensal strains that have been depleted. Identifying commensal bacterial species that have been lost by metagenomic sequencing; however, is time consuming and dependent on evolving sequencing and analytical platforms. We used ridge regression to identify commensal species missing from patients with reduced fecal butyrate and DCA concentrations (Figure 4). This model had an area under the curve of 93% and 91% on both testing and training sets, respectively (Figure 4A) and identified multiple species capable of producing butyrate as important features of samples with butyrate/DCA concentrations associated with improved clinical outcomes (Figure 4B). These species included *F. prausnitziiI*, *E. rectale,* and *A. hadrus* as well as many *Bifidobacterium* species, which are known to deconjugate conjugated BAs. Ridge regression of an independent cohort of 191 patients admitted to the MICU with diverse illnesses also identified some overlapping bacterial species that correlated with fecal butyrate and DCA concentrations (Figure A9).

## Discussion

Although intestinal microbiome composition is a well-established correlate of liver disease progression and outcomes, its role as a causative factor in human disease remains more speculative than proven. Clinical trials of microbiome augmentation with missing commensal microbes will be required to demonstrate causation but are challenging, in part because clinical tests to identify patients with microbiome deficiencies are yet to be developed. Herein we demonstrate that rapid LC-MS/MS quantitation of butyrate and DCA identifies patients, independent of MELD score and stage of liver disease, at risk for death. Our results suggest that same day quantitation of fecal metabolites can identify patients who might benefit from pre-emptive interventions to prevent complications of cirrhosis or who might be enrolled in clinical trials of microbiome-targeting therapies designed to restore missing organisms and lost microbially derived metabolites.

Metagenomic composition correlates with disease progression and risk of complications in many diseases, including cirrhosis.^1^^,^^2^^,^^4^^,^^5^^,^^7^^,^^20^^,^^26^^,^^28^, ^29^, ^30^, ^31^ While studies have identified “signatures” of different stages of liver disease, specific implicated taxa differ between studies. This may be due to differences in sequencing techniques (eg 16S vs metagenomic sequencing), evolving analytic techniques,^32^, ^33^, ^34^, ^35^, ^36^ or changing taxonomic nomenclature,^37^ which makes generalizing metagenomic findings difficult. On the other hand, microbially derived metabolites are well-defined chemical structures that mass-spectrometry can reproducibly quantify. Small studies have reported loss of butyrate and secondary BAs in patients with cirrhosis.^4^^,^^7^^,^^16^^,^^17^ In our large patient cohort, butyrate and DCA quantification identifies patients at increased risk of rehospitalization and death more accurately than metagenomic determination of the microbiome’s microbial composition.

Metabolomic profiling provides a reproducible snapshot of microbiome function and its potential impact on disease trajectory. Butyrate is a primary nutrient for enterocytes that enhances intestinal mucosal epithelial barrier functions and regulates T cell homeostasis.^38^, ^39^, ^40^ DCA, a secondary BA, is commonly thought of as a marker of a healthy microbiome but has been reported to have both proinflammatory and anti-inflammatory effects.^41^^,^^42^ As rifaximin administration results in clinical improvement but further reduces secondary BA levels, the implication of lower fecal secondary BA levels in cirrhosis also remains unclear.^4^^,^^16^ Secondary BA are necessary intermediates to generate modified secondary BA (eg 3-oxo-LCA, alloisoLCA, and isodeoxycholic acid), which are found at higher levels in healthy individuals and modulate adaptive immune responses.^43^^,^^44^ Simultaneous quantification of butyrate and DCA also provides information about microbiome composition that may be useful in therapeutic design. Using logistic regression, we identify multiple bacterial species that predict high levels of butyrate and DCA. Conversely, the presence of Enterobacteraceae species and vancomycin resistant-Enterococcus, which commonly infect patients with cirrhosis, predict low levels of butyrate and DCA. Butyrate, DCA and modified secondary BA also shape microbiome composition via anti-microbial effects that prevent intestinal colonization and expansion of pathogens, including multidrug resistant Enterobacteriaceae,^45^ vancomycin resistant-Enterococcus,^4^ and C. difficile,^44^^,^^46^ which predispose to infections and poor outcomes in patients with cirrhosis.

While some groups have used combinations of both targeted and untargeted serum metabolites to predict patient outcomes,^47^, ^48^, ^49^, ^50^, ^51^ subsequent intervention beyond risk stratification is unclear. We rapidly identify patients with low fecal butyrate and DCA concentrations, which associate with adverse outcomes. These findings lead to the testable hypothesis that microbiome reconstitution with bacteria that produce butyrate and DCA may improve clinical outcomes, but this association will need to be tested in future clinical trials. To date, limited studies have used fecal microbiota transplantation to treat various complications of liver diseases.^10^, ^11^, ^12^, ^13^^,^^22^ Unfortunately, concerns around safety and reproducibility limit clinical acceptance.^52^ Others have administered probiotics to patients with liver disease,^23^^,^^53^, ^54^, ^55^, ^56^, ^57^ but these are either individual strains or multiple strains with overlapping function with regard to acid production and bile salt deconjugation. Administering a more complex group of species will be important to reestablish lost functions, including butyrate production and sequential steps of BA metabolism. Optimal combinations, doses and timing of commensal bacteria for engraftment, metabolite production and clinical utility will need to be tested in future trials.^25^^,^^58^

Our study has several limitations. While metabolite analysis takes a step toward providing mechanistic insight, our observational study design limits our ability to determine causation. Single-center data limit generalizability, but our metagenomic findings are consistent with prior taxonomic analyses.^2^^,^^3^ Additionally, loss of beneficial metabolites has been observed in patient populations outside of liver disease. Our study cohort is heterogeneous with regards to disease etiology and severity, and patients are receiving treatments that could simultaneously impact microbiome composition and function as well as disease outcomes. For example, broad-spectrum antibiotics are commonly prescribed to patients with hospitalized with liver disease and deplete commensal organisms, exposure to antibiotics was not associated with worse outcomes in our analysis. These therapies are commonly prescribed and are often guideline recommended, so analyzing patients who have received antibiotics is necessary and offers an opportunity to study microbiome reconstitution and reduce rates of complications of advanced liver disease.

## Conclusion

We report that measurement of two fecal metabolites—butyrate and DCA—identifies patients with liver disease who are at increased risk of poor outcomes. These measurements can be used to identify patients who may benefit from microbiome therapy and can also be used to guide live biotherapeutic design for future clinical trials of rational microbiome reconstitution.

## Supplementary Materials

Figure A1 Incremental process to dichotomize continuous metagenomic variable for survival analysis. Survival analysis was performed on the cohort using incrementally increasing thresholds for invSimp from 1 to 32. (A, top) The number of samples that were classified as “high” (blue) and “low” (red) alpha-diversity, (A, middle) The difference between restricted mean survival time, and (A, bottom) the log_10_p-value is shown for each invSimp cut point. (B) Bootstrapping was performed for 1,000 iterations using cutpoint of invSimp = 4 with count vs. p-value plotted. 956 of the 1,000 bootstrapping iterations had *P* < .05.

Figure A2 Antibiotic and acid suppression exposure alters microbiome composition but is not associated with mortality. Fecal samples from 307 patients with liver disease and 27 healthy donors were analyzed by shotgun metagenomics. (A) Relative taxa abundance was calculated with MetaPhlan4. Samples are arranged in order of increasing invSimp within each group. Exposure to antibiotics and acid suppression within 7 days of sample collection is shown below each sample. (B and C) invSimp values are plotted by liver disease stage and stratified by any preceding (B) antibiotic or (C) acid suppression exposure. Each point represents a single value. Median and IQR are indicated by the line and box, respectively. Statistical comparisons were made between exposed and unexposed patients within a given disease stage using using Wilcoxin rank sum. P-values are as follows: ∗, *P* < .05; ∗∗, *P* < .01; ∗∗∗, *P* < .001; ∗∗∗∗, *P* < .0001. (D through G) Kaplan-Meier curves stratified by exposure to (D and E) antibiotics and (F and G) acid suppression for the (E and F) entire cohort and (E and G) only patients with decompensated cirrhosis. The number at risk is shown below.

Figure A3 Short chain fatty acids and bile acid metabolites associate with metagenomic alpha-diversity. Fecal samples from patients with liver disease (n = 307) and healthy donors (n= 27) were subjected to quantitative targeted metabolomics for bile acids (LC-MS) and short chain fatty acids (GC-MS). Samples were categorized as either high or low alpha-diversity (threshold invSimp = 4) for comparison. (A) Metabolites are plotted on a volcano plot (log_2_fold change vs. log_10_p-value) comparing fecal samples with high (blue) and low (red) alpha-diversity. P-values are corrected for multiple comparisons. Values with log2 fold-change > 1 (corresponding to a 2-fold change with a *P* < .05) were considered significant. (B) Spearman correlation coefficients were calculated for each of the 14 SCFA and BA metabolites when correlating with the levels of each of the other metabolites. Blue indicates a positive correlation and red indicates a negative correlation with darker shades representing stronger correlations. ∗, *P* < .05.

Figure A4 Antibiotics and exposure to acid suppressants alter fecal metabolite concentrations. Metabolite concentrations are plotted by liver disease stage and stratified by any preceding (A and B) antibiotic or (C and D) acid suppression exposure for butyrate (A and C) and DCA (B and D). Each point represents a single value. Median and IQR are indicated by the line and box, respectively. Statistical comparisons were made between exposed and unexposed patients within a given disease stage using using Wilcoxin rank sum. P-values are as follows: ∗, *P* < .05; ∗∗, *P* < .01; ∗∗∗, *P* < .001; ∗∗∗∗, *P* < .0001.

Figure A5 Butyrate production pathway genetic completion correlates with liver disease stage. (A) Genes implicated in butyrate production were classified by the pathway they are present in, including acetyl CoA (red), lysine (blue), glutarate (yellow) and 4-aminobutyrate (green) were queried. A shaded box indicates that a gene is included in a production pathway. (B) Overall completeness was calculated as the highest level of completeness of any single genetic pathway within the sample. The bar graph depicts the percent of samples from each stage of liver disease with at least one pathway with 100% overall completeness (green). (C) Fecal samples are arranged in order of increasing butyrate concentration as done in Figure 3. The copies per megabase (CoPM) of each gene involved in butyrate production is shown (C, top panel). Pathway completeness is graphed as a percentage of pathway genes detected in each sample. The highest percentage completeness for any set of genes implicated in a given pathway is graphed. Overall completeness (bottom, purple row) was calculated as the highest level of completeness of any genetic pathway within the sample.

Figure A6 Bioactive modified secondary bile acid concentrations correlate with liver disease stage. Iso-deoxycholic acid, alloiso-lithocholic acid and 3-oxo-lithocholic acid were quantified for fecal samples from patients with liver disease (n = 307) and healthy donors (n = 27). Fecal samples are grouped by stage of liver disease arranged in order of increasing iso-deoxycholic acid. Metabolite concentrations in μM are graphed in the top panels. Gene copies per megabase (CoPM) were calculated for bile salt hydroxysteroid dehydrogenase and reductase genes and are graphed in the bottom panel.

Figure A7 Microbiome characteristics at discharge and rehospitalization. The closest sample within 3 days of hospital discharge was analyzed from patients who survived their initial hospitalization without a liver transplant. Of the 307 patients enrolled in this study who produced a fecal sample, 238 were discharged from the hospital without a liver transplant. (A) There were 193 patients with a sample collected ≤ 3 days to discharge date, and 5 were excluded due to prior admissions. (B) From these 188 patients, there were 55 30-day re-hospitalizations or out of hospital deaths, shown in the red box. (C and D) Kaplan-Meier curves stratified by discharge sample alpha-diversity (threshold invSimp = 4) or butyrate and DCA concentrations (butyrate threshold = 2.5mM; DCA threshold = 40 μM) were generated for 30-day re-hospitalization. The number at risk at each time-point is shown below.

Figure A8 Incremental process to dichotomize continuous metabolomic variables for survival analysis. Fecal butyrate and DCA concentrations are continuous variables without defined thresholds to differentiate “high” and “low.” To determine an appropriate threshold, we performed survival analysis on the cohort using incrementally increasing thresholds for (A-C) butyrate and (D-F) DCA. For butyrate, increments of 100 were used from 0 to 10,000uM, and for DCA, increments of 10uM were used from 0 to 1,000uM. (A and D, top) The number of patients with fecal samples that were classified as “high” (blue) and “low” (red) metabolite levels for each threshold is shown in the top panel. (A and D, middle) The difference between restricted mean survival time is shown in the middle panel, and (bottom) the log_10_p-value is shown for each cut point in the bottom panel. There is a trend towards decreasing RMST difference and p-values with increasing butyrate and DCA thresholds. (B and E) After choosing a threshold of 2,500uM for butyrate and 40uM for DCA, we performed 1,000 iterations of bootstrapping to determine whether the observed differences and statistical significance could have happened by random chance. For butyrate 999 of the 1,000 iterations had *P* < .05, and for DCA all 1,000 iterations had *P* < .05. (C and F) Kaplan-Meier curves stratified by initial sample metabolite level (C, butyrate; F, DCA) were generated for 30-day death/transplant. The number at risk at each time-point is shown below.

Figure A9 Rapid metabolite measurement correlates with microbiome composition in an independent cohort of ICU patients. Ridge regression was performed with 80% training and 20% test sets with 5-fold, 2-repeat cross validation on the training set. This regression model used species level taxonomy as the input to distinguish between samples with high metabolite levels (butyrate ≥ 2.5mM and DCA ≥ 40uM) and low metabolite levels in an independent cohort of 191 patients hospitalized to the medical intensive care unit (MICU). (A) From this regression model, the mean area under the receiver operator curve had an area of 0.82 for training and 0.94 for testing. (B) The importance of 40 species (top and bottom 20 species) to the Ridge regression model is graphed for both training (blue) and testing (green). Negative values indicate that high species abundance is associated with samples with low metabolite levels, and positive values indicate that high species abundance is important feature of samples with high metabolite levels.
