# Supplementary figures and images for: Fecal Butyrate and Deoxycholic Acid Concentrations Correlate With Mortality in Patients With Liver Disease
Source: Gastro Hep Adv. 2025 May 9;4(8):100695. doi: 10.1016/j.gastha.2025.100695 (PMC12197998; doi:10.1016/j.gastha.2025.100695)

A

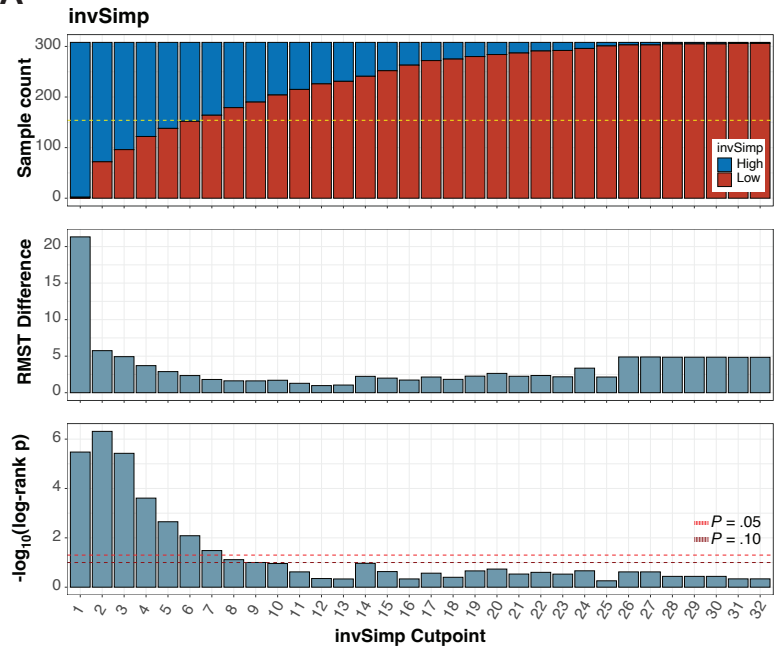

B

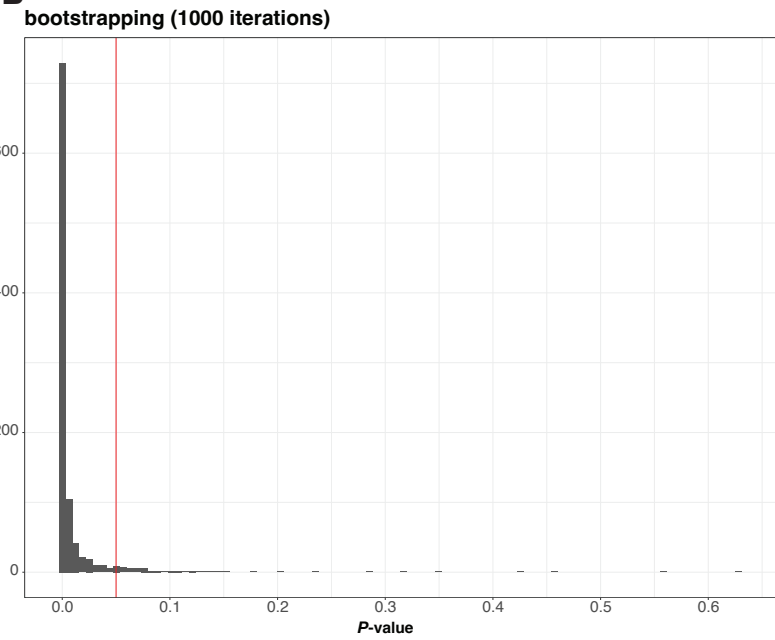

Supplement: Figure A1 — Incremental process to dichotomize continuous metagenomic variable for survival analysis. Survival analysis was performed on the cohort using incrementally increasing thresholds for invSimp from 1 to 32. (A, top) The number of samples that were classified as “high” (blue) and “low” (red) alpha-diversity, (A, middle) The difference between restricted mean survival time, and (A, bottom) the log10p-value is shown for each invSimp cut point. (B) Bootstrapping was performed for 1,000 iterations using cutpoint of invSimp = 4 with count vs. p-value plotted. 956 of the 1,000 bootstrapping iterations had P < .05. [file mmc1.pdf]

FIGURE S2

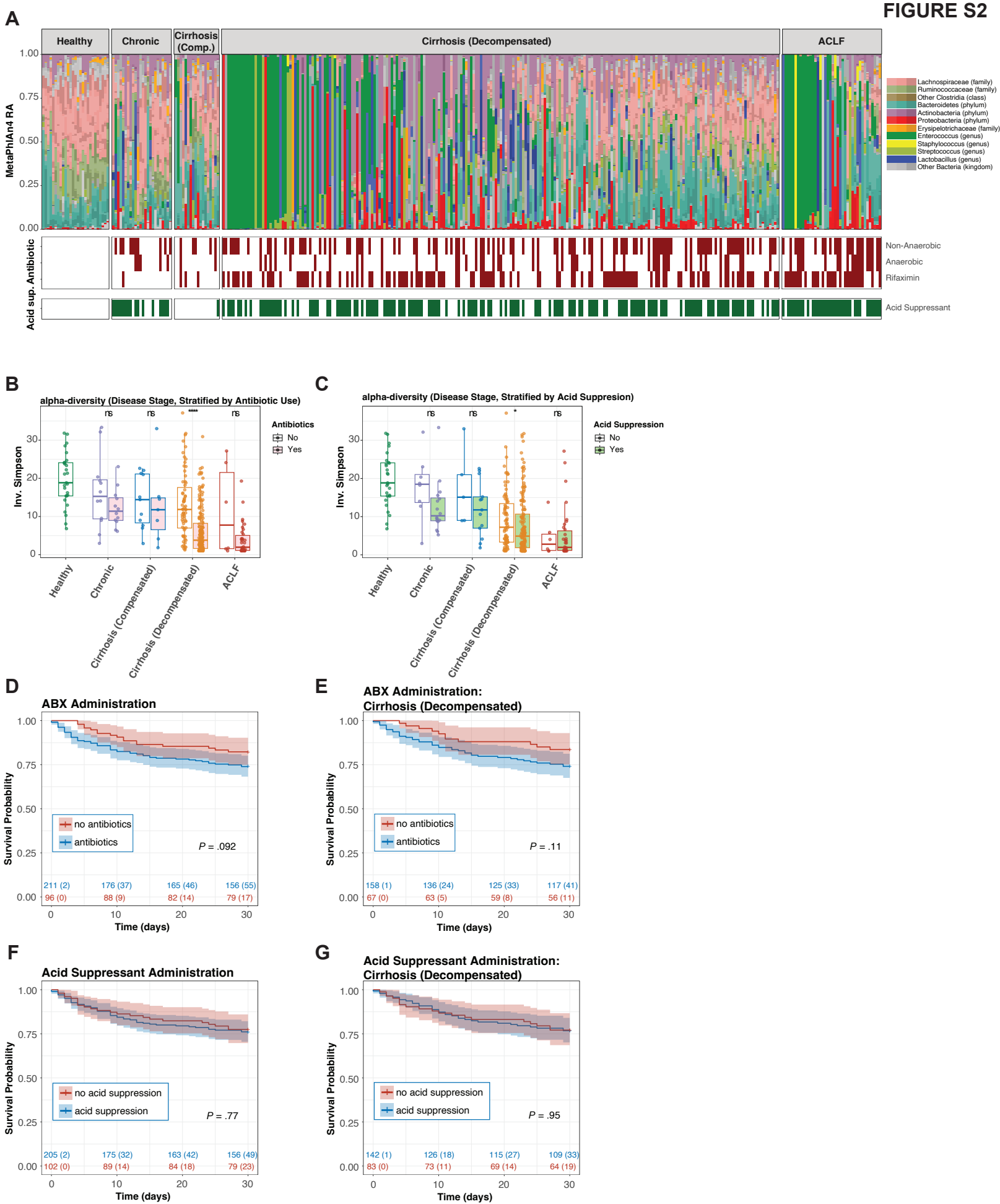

Supplement: Figure A2 — Antibiotic and acid suppression exposure alters microbiome composition but is not associated with mortality. Fecal samples from 307 patients with liver disease and 27 healthy donors were analyzed by shotgun metagenomics. (A) Relative taxa abundance was calculated with MetaPhlan4. Samples are arranged in order of increasing invSimp within each group. Exposure to antibiotics and acid suppression within 7 days of sample collection is shown below each sample. (B and C) invSimp values are plotted by liver disease stage and stratified by any preceding (B) antibiotic or (C) acid suppression exposure. Each point represents a single value. Median and IQR are indicated by the line and box, respectively. Statistical comparisons were made between exposed and unexposed patients within a given disease stage using using Wilcoxin rank sum. P-values are as follows: ∗, P < .05; ∗∗, P < .01; ∗∗∗, P < .001; ∗∗∗∗, P < .0001. (D through G) Kaplan-Meier curves stratified by exposure to (D and E) antibiotics and (F and G) acid suppression for the (E and F) entire cohort and (E and G) only patients with decompensated cirrhosis. The number at risk is shown below. [file mmc2.pdf]

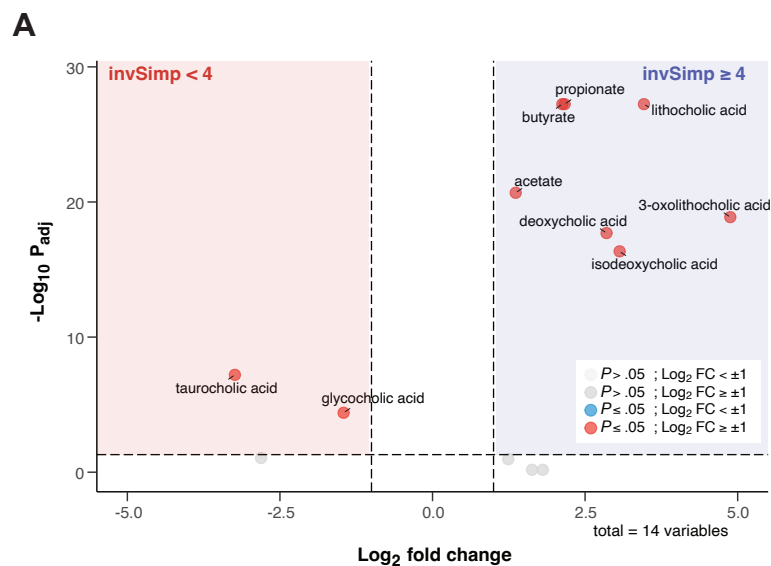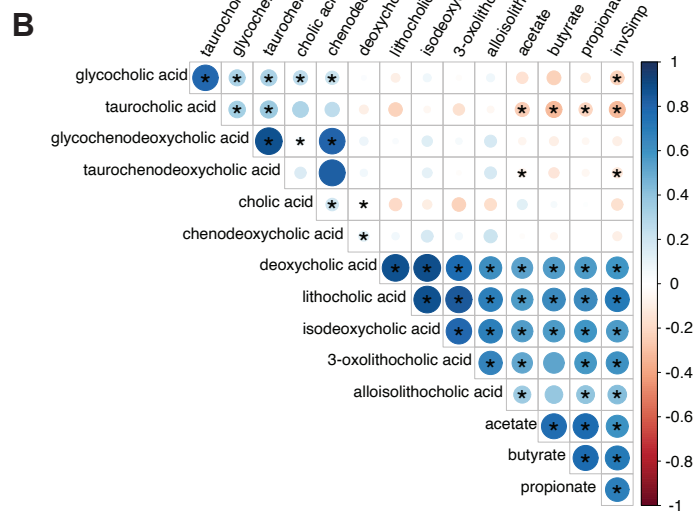

Supplement: Figure A3 — Short chain fatty acids and bile acid metabolites associate with metagenomic alpha-diversity. Fecal samples from patients with liver disease (n = 307) and healthy donors (n= 27) were subjected to quantitative targeted metabolomics for bile acids (LC-MS) and short chain fatty acids (GC-MS). Samples were categorized as either high or low alpha-diversity (threshold invSimp = 4) for comparison. (A) Metabolites are plotted on a volcano plot (log2fold change vs. log10p-value) comparing fecal samples with high (blue) and low (red) alpha-diversity. P-values are corrected for multiple comparisons. Values with log2 fold-change > 1 (corresponding to a 2-fold change with a P < .05) were considered significant. (B) Spearman correlation coefficients were calculated for each of the 14 SCFA and BA metabolites when correlating with the levels of each of the other metabolites. Blue indicates a positive correlation and red indicates a negative correlation with darker shades representing stronger correlations. ∗, P < .05. [file mmc3.pdf]

FIGURE S4

A

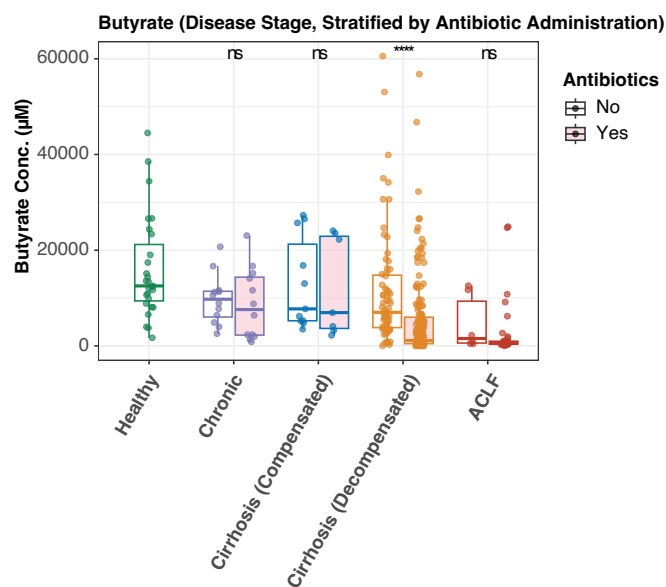

B

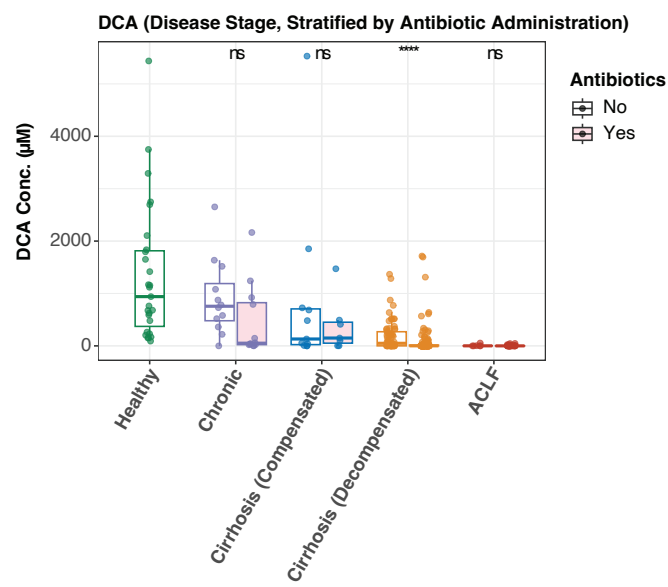

C

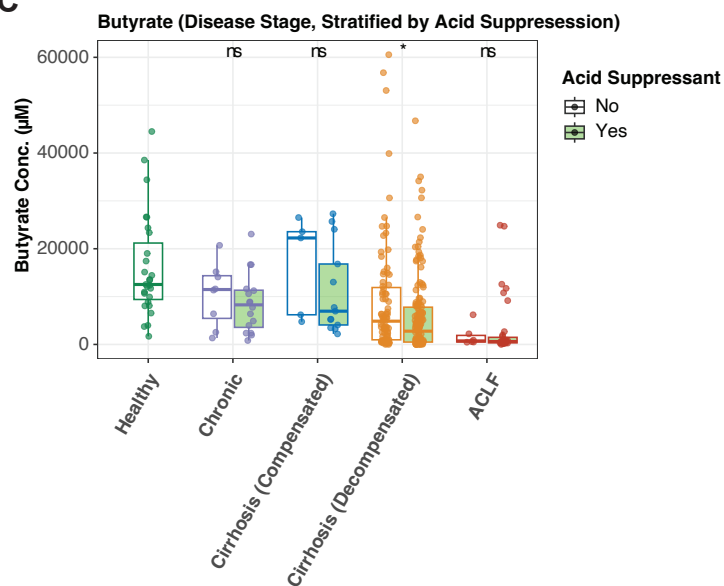

D

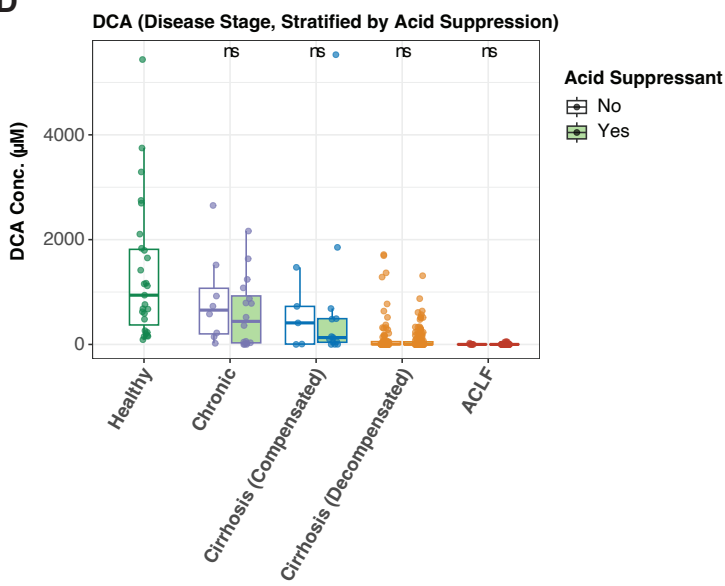

Supplement: Figure A4 — Antibiotics and exposure to acid suppressants alter fecal metabolite concentrations. Metabolite concentrations are plotted by liver disease stage and stratified by any preceding (A and B) antibiotic or (C and D) acid suppression exposure for butyrate (A and C) and DCA (B and D). Each point represents a single value. Median and IQR are indicated by the line and box, respectively. Statistical comparisons were made between exposed and unexposed patients within a given disease stage using using Wilcoxin rank sum. P-values are as follows: ∗, P < .05; ∗∗, P < .01; ∗∗∗, P < .001; ∗∗∗∗, P < .0001. [file mmc4.pdf]

FIGURE S5

A

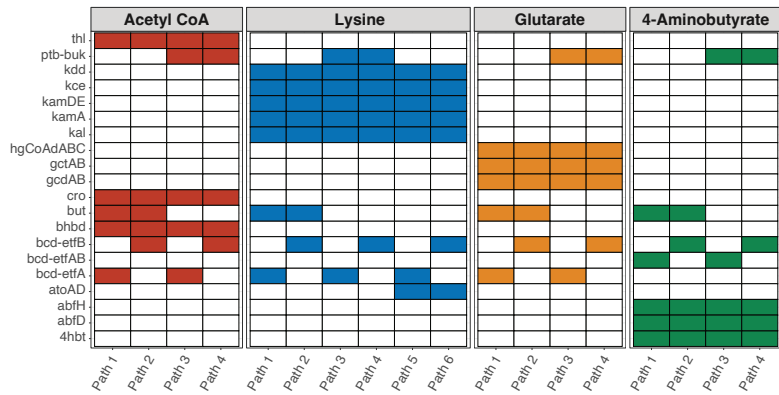

B

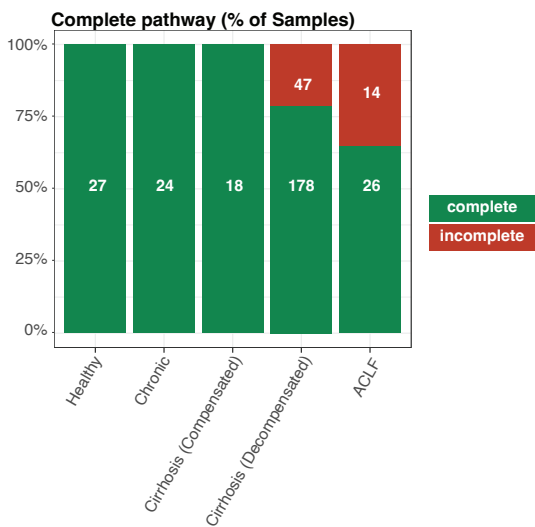

C

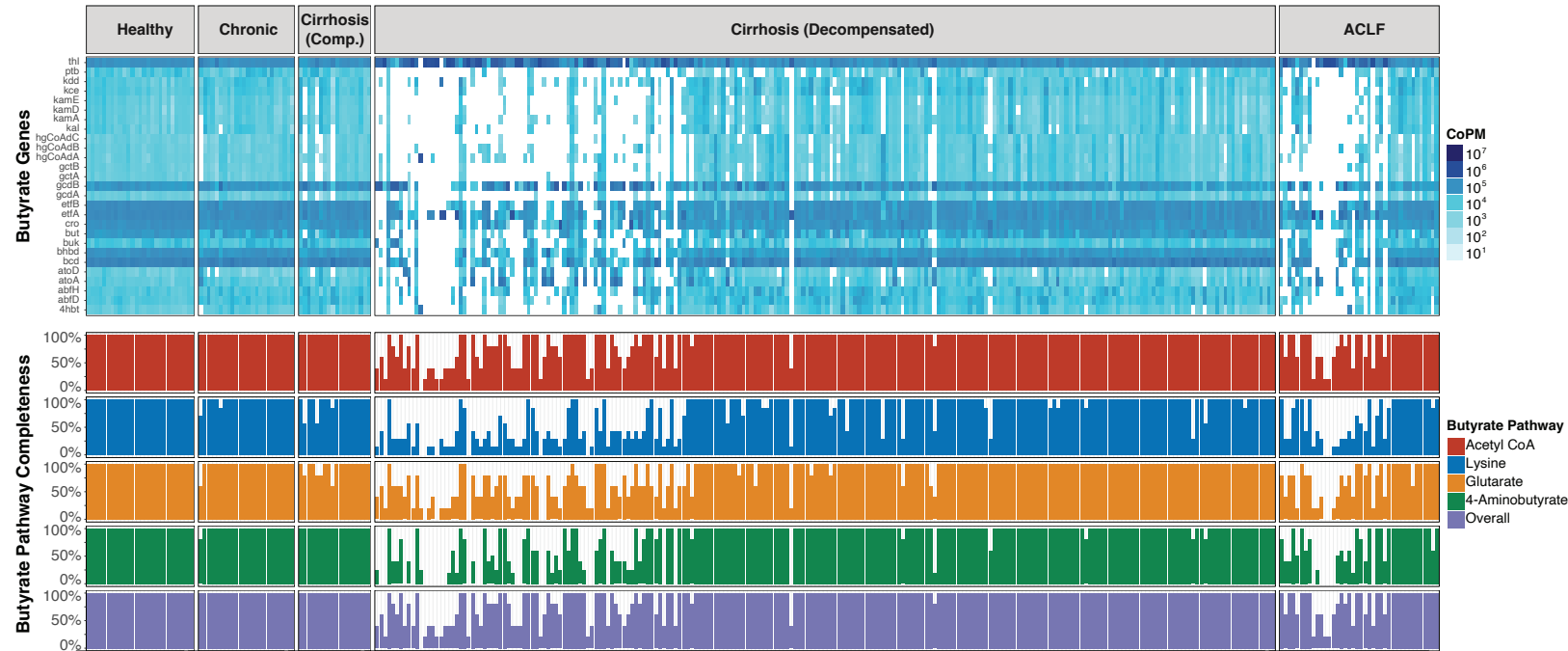

Supplement: Figure A5 — Butyrate production pathway genetic completion correlates with liver disease stage. (A) Genes implicated in butyrate production were classified by the pathway they are present in, including acetyl CoA (red), lysine (blue), glutarate (yellow) and 4-aminobutyrate (green) were queried. A shaded box indicates that a gene is included in a production pathway. (B) Overall completeness was calculated as the highest level of completeness of any single genetic pathway within the sample. The bar graph depicts the percent of samples from each stage of liver disease with at least one pathway with 100% overall completeness (green). (C) Fecal samples are arranged in order of increasing butyrate concentration as done in Figure 3. The copies per megabase (CoPM) of each gene involved in butyrate production is shown (C, top panel). Pathway completeness is graphed as a percentage of pathway genes detected in each sample. The highest percentage completeness for any set of genes implicated in a given pathway is graphed. Overall completeness (bottom, purple row) was calculated as the highest level of completeness of any genetic pathway within the sample. [file mmc5.pdf]

FIGURE S6

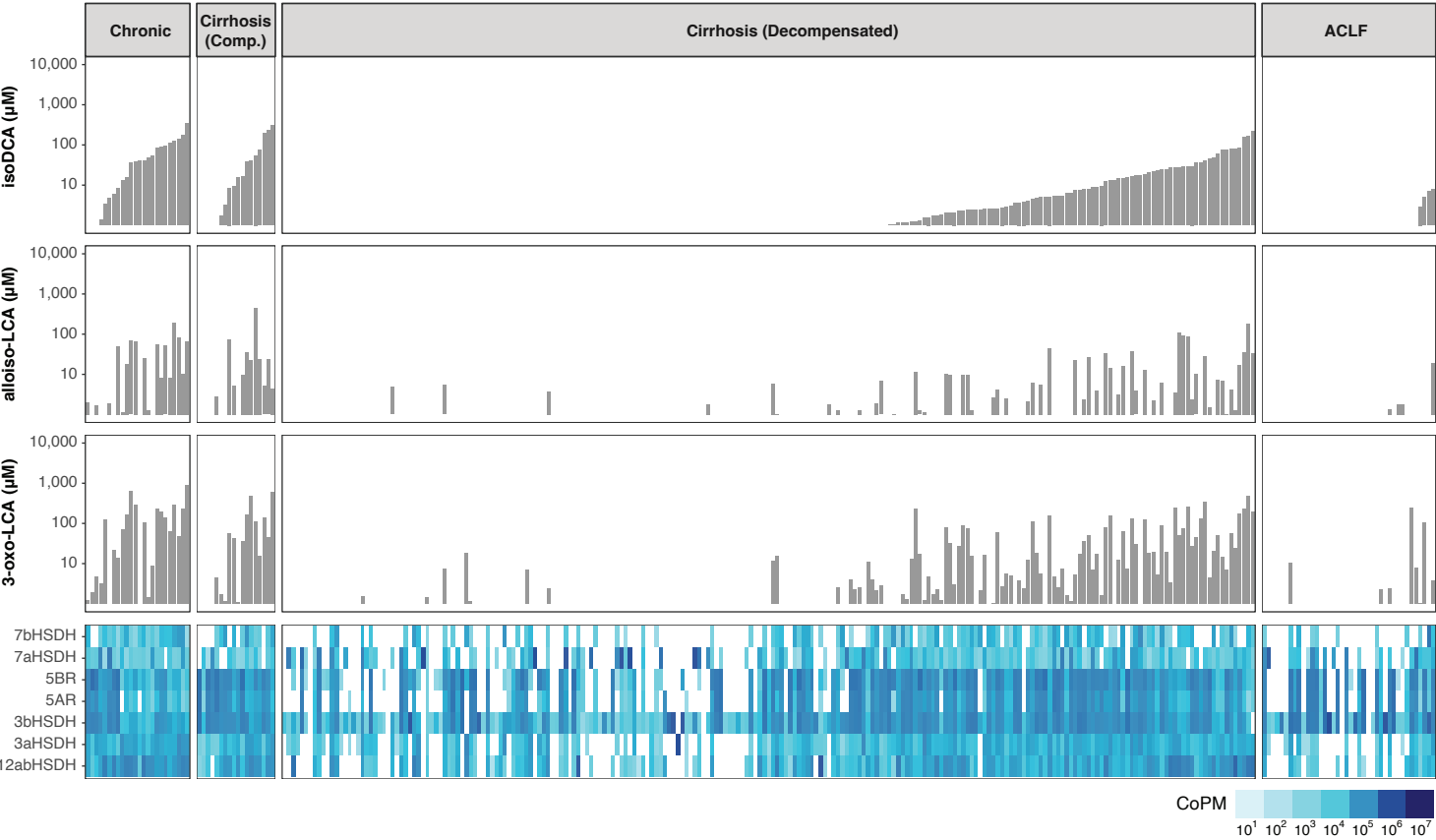

Supplement: Figure A6 — Bioactive modified secondary bile acid concentrations correlate with liver disease stage. Iso-deoxycholic acid, alloiso-lithocholic acid and 3-oxo-lithocholic acid were quantified for fecal samples from patients with liver disease (n = 307) and healthy donors (n = 27). Fecal samples are grouped by stage of liver disease arranged in order of increasing iso-deoxycholic acid. Metabolite concentrations in μM are graphed in the top panels. Gene copies per megabase (CoPM) were calculated for bile salt hydroxysteroid dehydrogenase and reductase genes and are graphed in the bottom panel. [file mmc6.pdf]

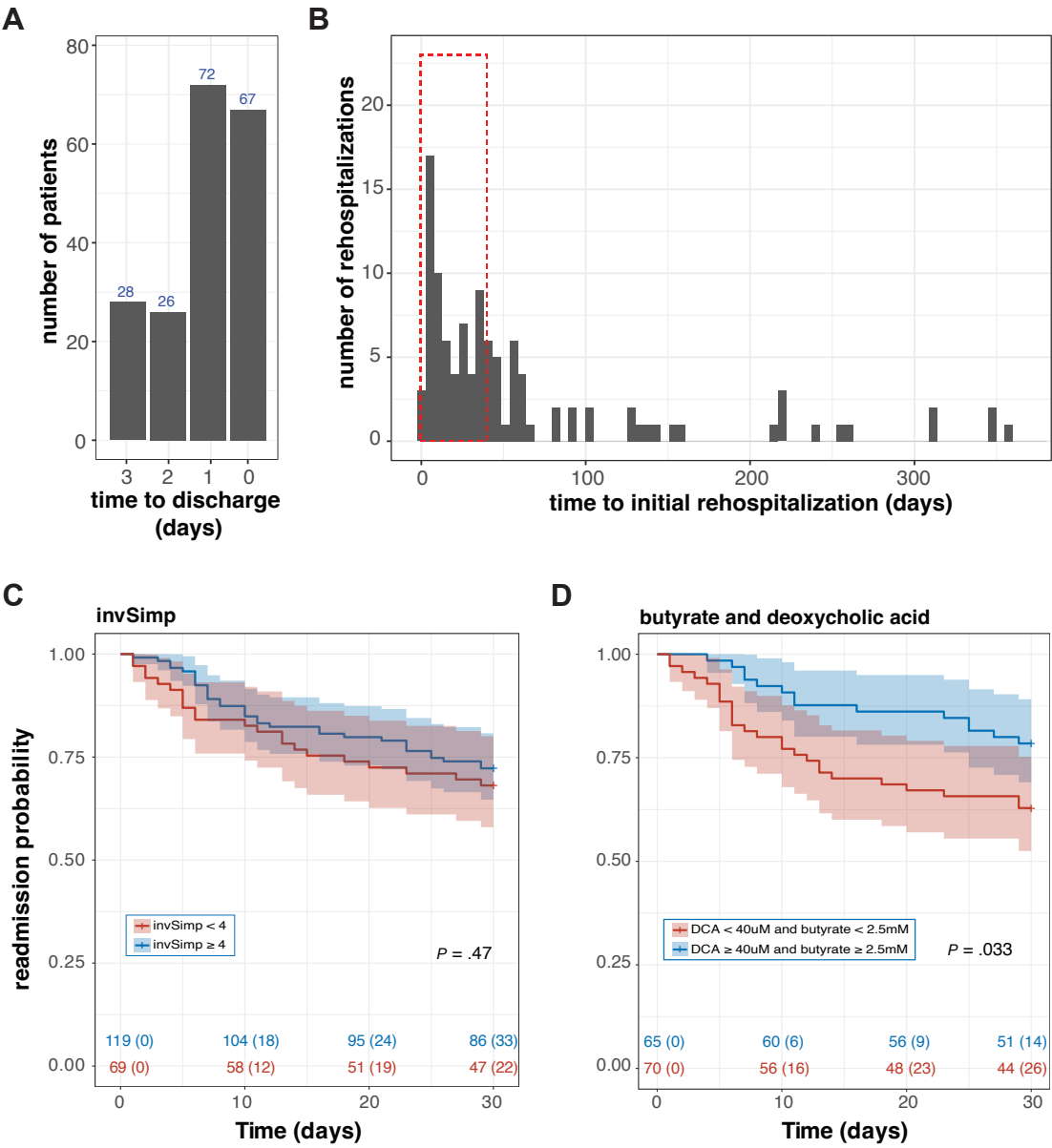

Supplement: Figure A7 — Microbiome characteristics at discharge and rehospitalization. The closest sample within 3 days of hospital discharge was analyzed from patients who survived their initial hospitalization without a liver transplant. Of the 307 patients enrolled in this study who produced a fecal sample, 238 were discharged from the hospital without a liver transplant. (A) There were 193 patients with a sample collected ≤ 3 days to discharge date, and 5 were excluded due to prior admissions. (B) From these 188 patients, there were 55 30-day re-hospitalizations or out of hospital deaths, shown in the red box. (C and D) Kaplan-Meier curves stratified by discharge sample alpha-diversity (threshold invSimp = 4) or butyrate and DCA concentrations (butyrate threshold = 2.5mM; DCA threshold = 40 μM) were generated for 30-day re-hospitalization. The number at risk at each time-point is shown below. [file mmc7.pdf]

FIGURE S8

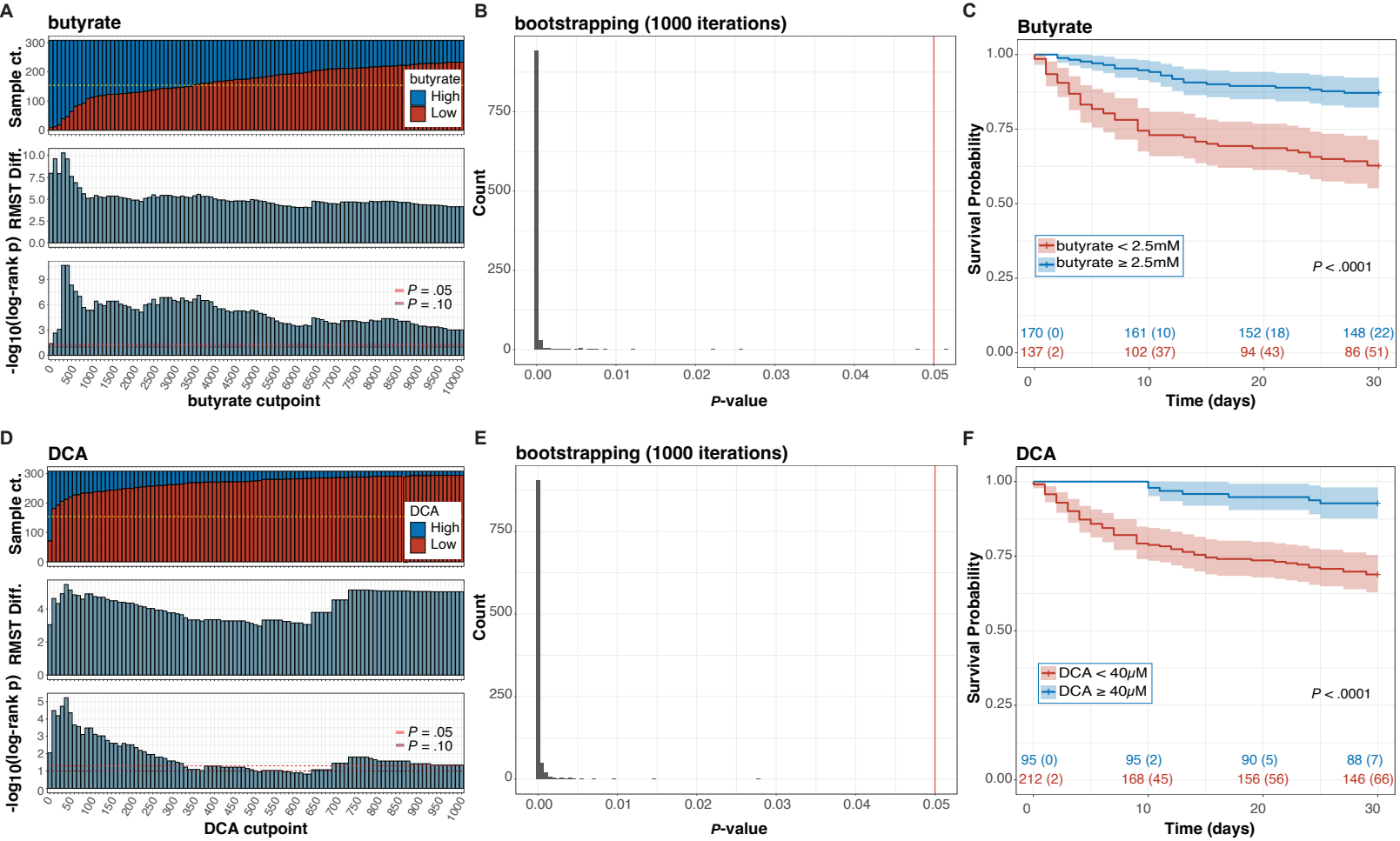

Supplement: Figure A8 — Incremental process to dichotomize continuous metabolomic variables for survival analysis. Fecal butyrate and DCA concentrations are continuous variables without defined thresholds to differentiate “high” and “low.” To determine an appropriate threshold, we performed survival analysis on the cohort using incrementally increasing thresholds for (A-C) butyrate and (D-F) DCA. For butyrate, increments of 100 were used from 0 to 10,000uM, and for DCA, increments of 10uM were used from 0 to 1,000uM. (A and D, top) The number of patients with fecal samples that were classified as “high” (blue) and “low” (red) metabolite levels for each threshold is shown in the top panel. (A and D, middle) The difference between restricted mean survival time is shown in the middle panel, and (bottom) the log10p-value is shown for each cut point in the bottom panel. There is a trend towards decreasing RMST difference and p-values with increasing butyrate and DCA thresholds. (B and E) After choosing a threshold of 2,500uM for butyrate and 40uM for DCA, we performed 1,000 iterations of bootstrapping to determine whether the observed differences and statistical significance could have happened by random chance. For butyrate 999 of the 1,000 iterations had P < .05, and for DCA all 1,000 iterations had P < .05. (C and F) Kaplan-Meier curves stratified by initial sample metabolite level (C, butyrate; F, DCA) were generated for 30-day death/transplant. The number at risk at each time-point is shown below. [file mmc8.pdf]

A

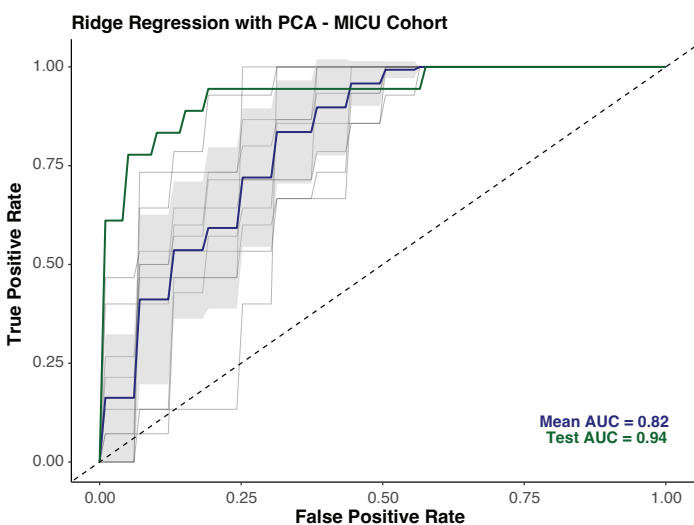

B

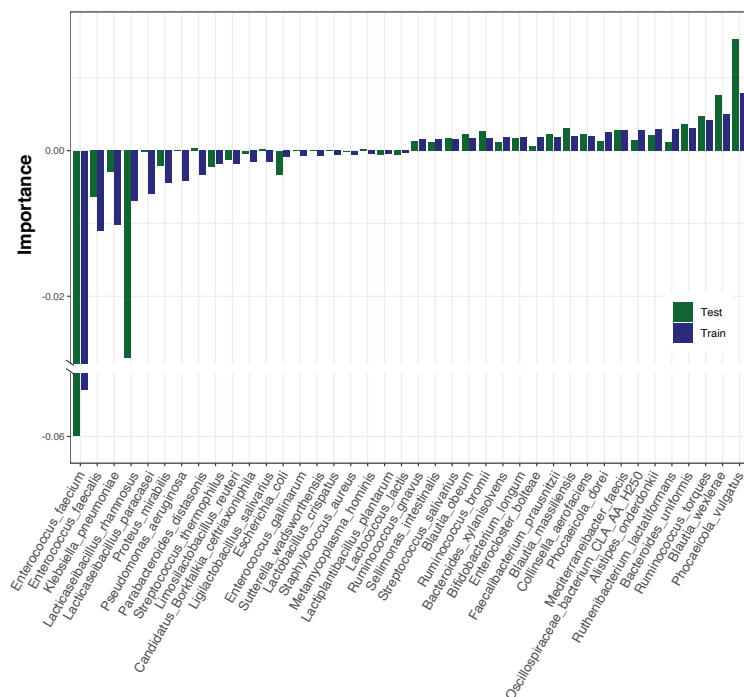

C

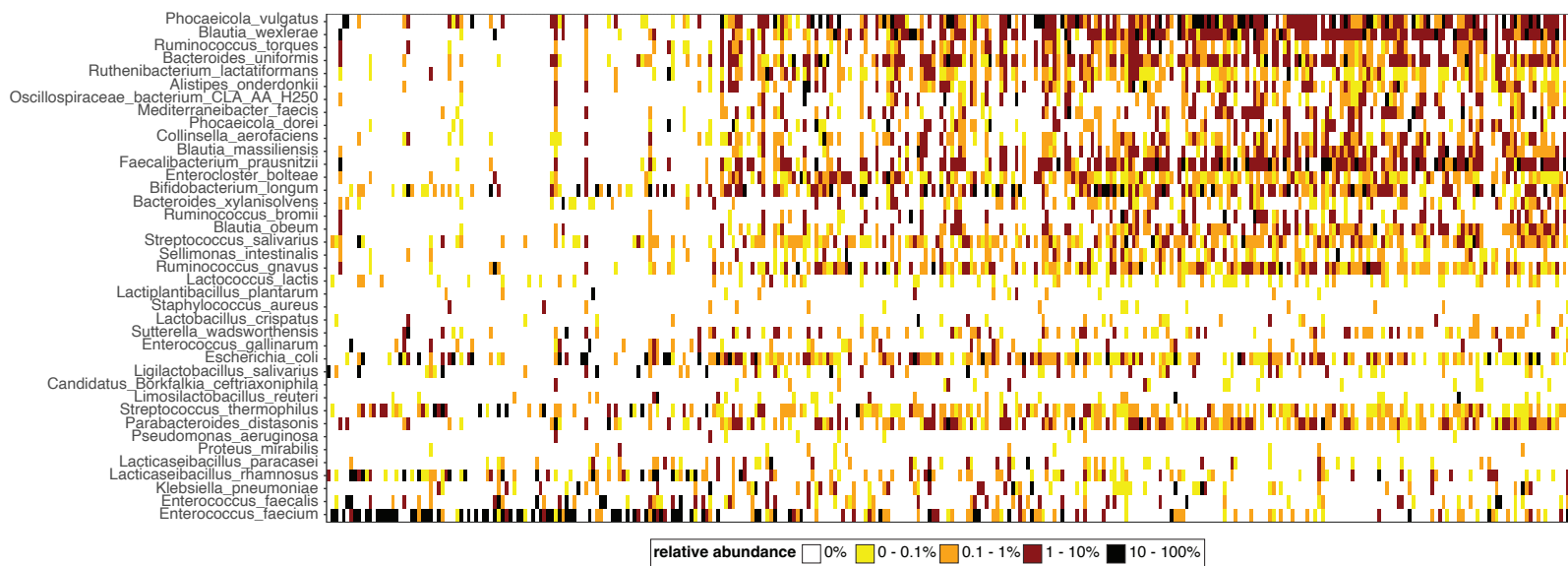

Supplement: Figure A9 — Rapid metabolite measurement correlates with microbiome composition in an independent cohort of ICU patients. Ridge regression was performed with 80% training and 20% test sets with 5-fold, 2-repeat cross validation on the training set. This regression model used species level taxonomy as the input to distinguish between samples with high metabolite levels (butyrate ≥ 2.5mM and DCA ≥ 40uM) and low metabolite levels in an independent cohort of 191 patients hospitalized to the medical intensive care unit (MICU). (A) From this regression model, the mean area under the receiver operator curve had an area of 0.82 for training and 0.94 for testing. (B) The importance of 40 species (top and bottom 20 species) to the Ridge regression model is graphed for both training (blue) and testing (green). Negative values indicate that high species abundance is associated with samples with low metabolite levels, and positive values indicate that high species abundance is important feature of samples with high metabolite levels. [file mmc9.pdf]
