# Supplementary material for: Fecal Butyrate and Deoxycholic Acid Concentrations Correlate With Mortality in Patients With Liver Disease
Source: Gastro Hep Adv. 2025 May 9;4(8):100695. doi: 10.1016/j.gastha.2025.100695 (PMC12197998; doi:10.1016/j.gastha.2025.100695)
Supplement: Supplementary Material [file mmc10.docx]

**SUPPLEMENTAL METHODS**

*Metagenomic Analysis*

Metagenomic profiles of all clinical stool samples were generated using a bioBakery workflow executed within the Nextflow-based Workflow Orchestration Service on the University of Chicago’s HPC cluster. A custom Nextflow script was employed to execute the bioBakery workflow using the “dfiuchicago/shotgun:2.1.2” Docker container. Initial quality control and contamination removal were performed with “KneadData v0.10.0", using the human genome reference “hg37dec_v0.1.1”. Cleaned reads were analyzed with MetaPhlAn (v4.1.1) 1 and the database “mpa_vJun23_CHOCOPhlAnSGB_202403". Default parameters were applied, with the exceptions of enabling the “unclassified_estimation” and “rel_ab_w_read_stats” flags to enhance taxonomic profiling and relative abundance statistics. In addition, high-quality reads are queried against genes of interest, such as butyrate production genes and bile salt metabolism genes, using DIAMOND (v2.0.4),^28^ and hits are filtered with threshold > 80% identity, > 80% protein coverage, then abundance is tabulated into counts per million mapped reads. Raw relative abundance values were first normalized to counts per million (CPM) by scaling to a sum of 1,000,000 for each sample. Alpha-diversity of fecal samples was estimated using the Inverse Simpson Index using the Vegan package in R.

Manually curated sets of protein sequences for genes associated with bile acid^29–33^ and butyrate (FASTA file; http://193.175.244.101/Butyrate) metabolism were created.^34^ To identify and quantify the abundance of bile acid and butyrate metabolism genes in metagenomic data, sequence similarity searches were performed with DIAMOND v2.1.8.^35^ Custom reference databases were created from manually curated sets of protein sequences for both bile acid and butyrate metabolism genes using the DIAMOND makedb command. Paired-end metagenomic reads were aligned against these reference databases using DIAMOND blastx with default parameters. Alignment results were filtered using stringent criteria (minimum 80% sequence identity and E-value cutoff of 1e-3) to ensure high-confidence matches. Only the highest-scoring alignment (based on bitscore) was retained for each query sequence to avoid multiple counting. Gene abundance was normalized using the transcripts per million (TPM) method to account for gene length and sequencing depth. This involved calculating reads per kilobase (RPK) by dividing raw read counts by gene length (in base pairs) multiplied by 1000, followed by scaling these values using a sample-specific scaling factor calculated as the sum of RPK values divided by 1,000,000.

*Supplemental Statistical Analysis*

*Dichotomization of continuous variables*

To determine relevant thresholds for continuous variables (alpha diversity and butyrate and DCA concentrations), we performed an optimal cutpoint analysis using survival outcomes from R packages survival (v3.7.0), survminer (v0.5.0), and ggsurvfit (v1.1.0). For each metric, we systematically evaluated multiple potential cutpoints while assessing their statistical significance and relevance to clinical outcomes. The analysis incorporated three key parameters: log-rank test p-values for survival difference between groups, restricted mean survival time (RMST) differences, and the resulting group size distributions. Survival times were censored at 30 days, with appropriate handling of missing values by imputation using mean values for the respective metrics. For each candidate cutpoint, samples were stratified into "High" and "Low" groups (i.e. above or below threshold), and survival differences were assessed using the log-rank test. To ensure balanced group sizes and clinical applicability, we monitored the distribution of samples across the High and Low groups for each cutpoint. Statistical significance was evaluated at both p < 0.05 and p < 0.10 thresholds. The optimal cutpoint for each metric was selected based on a combination of statistical significance (-log10 transformed p-values), maximal RMST difference, and balanced group sizes, visualized through a composite plot combining these three parameters as depicted in Figures S1 and S5. To ensure that statistical significance was not an effect of the specific set of patients, we performed bootstrapping with 50 random sets of the population.

*Cox proportional hazards*

Cox proportional hazards regression analyses were performed using the survival package in R to assess the relationship between mortality risk, microbiome parameters (alpha-diversity, butyrate and DCA levels) and known risk factors for mortality in liver disease (survival::coxph). For categorical analyses, the previously determined optimal cutpoints were used to stratify patients into high and low-risk groups for each metric.

*Volcano plot analyses*

Differential abundance analysis was performed to compare metabolite concentrations between high and low alpha diversity groups (defined by Inverse Simpson index cutoff). Samples were stratified based on a predetermined alpha diversity cutoff, and metabolite concentrations were compared between groups using Wilcoxon rank-sum tests. P-values were adjusted for multiple comparisons using the Benjamini-Hochberg method. The magnitude of change was calculated as log2 fold change between high and low diversity groups, with zero values replaced by 0.0001 to enable fold change calculations. Results were visualized using a modified volcano plot (EnhancedVolcano package R), displaying the relationship between statistical significance (-log10 adjusted p-value) and effect size (log2 fold change). Metabolites with absolute log2 fold change ≥ 1 and adjusted *P* < .05 were considered significantly different between groups. The visualization included custom annotations highlighting metabolites enriched in high and low diversity conditions, with significance thresholds marked.

*Random Forest Analysis*

An oblique random survival forest model was used to assess patient and microbiome characteristics that may affect survival. This was done as previously described using the R package aorsf^38^. Briefly, metagenomics taxon, metabolite compounds and important demographic and clinical variables were abundance filtered, variance filtered and normalized, then feed into the model using function aorsf::orsf with survival model of time-to-event and 30-day event as response variable. Negation importance was calculated using function: aorsf::orsf_vi_negate, and the top 30 features were chosen to further investigate in a volcano plot.

*Predicting Metabolite Profiles based on Microbiome data*

We developed a machine learning approach to predict metabolite profiles based on bacterial species abundance data derived from MetaPhlAn analysis. The initial dataset comprised 253 samples with abundance data for 878 bacterial species. To remove potential noise from rare species, a prevalence filter was applied, removing species that were present in fewer than 3 samples, which resulted in the removal of 295 species, leaving 583 species for analysis. Samples were classified into two groups based on previously determined survival-based cutoffs: 116 samples with high butyrate and high deoxycholic acid (DCA) levels and 137 samples with low levels of both metabolites. The input features consisted of bacterial relative abundance data (summing to 100% for each sample), with zero values retained to represent species absence. To reduce dimensionality and address potential collinearity, a Principal Component Analysis (PCA) was applied, retaining sufficient components to explain 95% of the total variance. The data was then split into training (80%) and test (20%) sets using stratified sampling to maintain class proportions. We then implemented a logistic regression classifier with hyperparameter optimization using grid search cross-validation. The hyperparameter grid explored various regularization methods (L1 [Lasso], L2 [Ridge], Elastic Net, and no regularization) with different solvers and regularization strengths ranging from 0.001 to 1000. Model performance was evaluated using repeated stratified k-fold cross-validation (5 folds, 2 repeats) to ensure robust performance estimation while minimizing potential bias from excessive resampling. The optimal model parameters were identified as L2 regularization (Ridge) with a regularization strength (C) of 10 using the newton-cg solver. The final model demonstrated strong predictive performance on the held-out test set, achieving an ROC-AUC of 0.904 and an accuracy of 0.804. We visualized the results using ROC curves showing both cross-validation performance (with standard deviation bands) and test set performance. To interpret the biological significance of the model, we calculated feature importance scores by projecting the logistic regression coefficients back to the original feature space through the PCA transformation matrix. Feature importance was calculated separately for training and test sets to assess the stability of feature rankings. For visualization and interpretation, we selected the top 20 most important features in each direction (positive and negative associations) based on their absolute importance values in the training set.
